# Adverse drug reactions related to methotrexate: a real-world pharmacovigilance study using the FAERS database from 2004 to 2024

**DOI:** 10.3389/fimmu.2025.1586361

**Published:** 2025-06-04

**Authors:** Siyu Liu, Zhang Yuan, Shicong Rao, Wenxing Li, Taiwei Wu, Shuzhen Deng, Yongying Zhong, Jinyu Lin, Wei Guo, Shiwei Yuan

**Affiliations:** Longyan Hospital of Xiamen University, School of Medicine, Xiamen University, Xiamen, China

**Keywords:** methotrexate, pharmacovigilance, adverse drug reactions, FAERS database, real world

## Abstract

**Objective:**

To comprehensively analyze the safety profile of Methotrexate in clinical use, clarify the incidence of adverse reactions and associated influencing factors, and provide evidence for safe medication practices in clinical settings.

**Methods:**

This study retrieved data from the FDA Adverse Event Reporting System (FAERS) database from the first quarter of 2004 to the fourth quarter of 2024. Data filtering was conducted on the main suspect drug. Data extraction and cleaning were performed using R software, and various statistical methods, including ROR (Reporting Odds Ratio), PRR (Proportional Reporting Ratio), BCPNN (Bayesian Confidence Propagation Neural Network), and MGPS (Multi-Item Gamma Poisson Shrinker), were employed to detect adverse drug reaction signals. Subgroup analyses based on gender, age, and reporter categories were performed to explore differences.

**Results:**

A total of 130,818 methotrexate (MTX)-related adverse event (AE) reports were included. Females accounted for 64.2% of reporters, with adults aged 18–64.9 years reporting the most. AEs primarily affected the immune, musculoskeletal, and hematologic systems. “General Disorders and Administration Site Conditions” was the most frequently reported system organ class [n=106,183, ROR (95% CI)=1.21 (1.21–1.22)], while “Immune System Disorders” showed the strongest signal [n=13,313, ROR (95% CI)=2.35 (2.31–2.39)]. Adverse reactions varied by gender and age: females were more likely to report Drug Hypersensitivity [n=6,192, ROR (95% CI)=4.69 (4.57–4.82)], while males reported Nausea more often [n=1,624, ROR (95% CI)=1.17 (1.12–1.23)]. Elderly patients (≥65 years) had an increased risk of drug hypersensitivity [n=2,894, ROR (95% CI)=7.91 (7.61–8.22)]. Reporting priorities differed: consumers frequently reported “Drug Ineffective” [n=5,729, ROR (95% CI)=2.24 (2.18–2.3)] and “Pain” [n=1,746, ROR (95% CI)=1.69 (1.61–1.77)], while healthcare professionals focused on DRUG INEFFECTIVE [n=9,982, ROR (95% CI)=4.16 (4.08–4.25)]. Additionally, the time to onset of MTX-induced AEs varied significantly across subgroups.

**Conclusion:**

This study reveals the safety characteristics of MTX in clinical use, confirms known adverse reactions, and identifies new potential adverse effects. It suggests that clinicians should enhance monitoring based on patient factors such as gender and age, particularly for immune system-related adverse reactions in elderly patients. Moreover, the spectrum of MTX’s side effects may be broader than previously recognized, warranting further research to ensure patient safety in drug use.

## 1 Introduction

Methotrexate (MTX) (1, 2) is a widely used antimetabolite in clinical practice, primarily exerting its antitumor, immunosuppressive, and anti-inflammatory effects through the inhibition of dihydrofolate reductase (DHFR) (3). As a folate analog, MTX competitively inhibits the activity of DHFR, thereby reducing the synthesis of tetrahydrofolate (THF) (4). THF is an essential coenzyme in DNA synthesis, repair, and methylation processes, playing a critical role in the synthesis of pyrimidines and purines. By inhibiting the formation of THF, MTX reduces the cell’s nucleotide synthesis capacity, ultimately blocking cell proliferation. Furthermore, MTX can influence the metabolism of homocysteine inside cells, induce oxidative stress, and further lead to apoptosis. Its antitumor effects primarily stem from this mechanism, which inhibits the proliferation of cancer cells (5), while also treating immune system diseases, such as rheumatoid arthritis, through immunosuppressive action (6, 7).

Currently, the use of MTX in various diseases has entered a more refined and personalized treatment stage. In cancer therapy, especially in the treatment of acute leukemia and lymphoma (8), the dosage adjustments and combination therapy strategies for MTX have gradually moved toward precision medicine. By monitoring patients’ DHFR gene expression and metabolic products (such as 7-OH-MTX), clinicians can develop personalized treatment plans for each patient, thereby improving treatment efficacy and minimizing unnecessary side effects. In the treatment of rheumatic and immune diseases, MTX, as one of the foundational treatments for rheumatoid arthritis, optimizes therapeutic effects not only through traditional drug dosage adjustments but also through safety monitoring. Since MTX was approved by the U.S. FDA in 1953, its applications have expanded to include treatments for acute lymphoblastic leukemia, breast cancer, severe psoriasis, rheumatoid arthritis, and juvenile rheumatoid arthritis. In fact, the success of MTX in treating multiple diseases has made it an essential resource in medicine, improving patient outcomes and alleviating the burden caused by disease progression. However, reports of MTX’s toxic side effects have been common in clinical practice since 1958 (9). Therefore, understanding the safety profile of MTX in clinical practice is crucial. According to prescribing information, the most common adverse reactions include ulcerative stomatitis, leukopenia, elevated transaminases, anemia, nausea, and other gastrointestinal symptoms (10–14). This study, utilizing data from the FDA Adverse Event Reporting System (FAERS), provides a comprehensive analysis of MTX’s real-world safety profile, offering valuable evidence for healthcare professionals in the application of MTX.

## 2 Materials and methods

### 2.1 Data sources and processing

**Figure 1:**
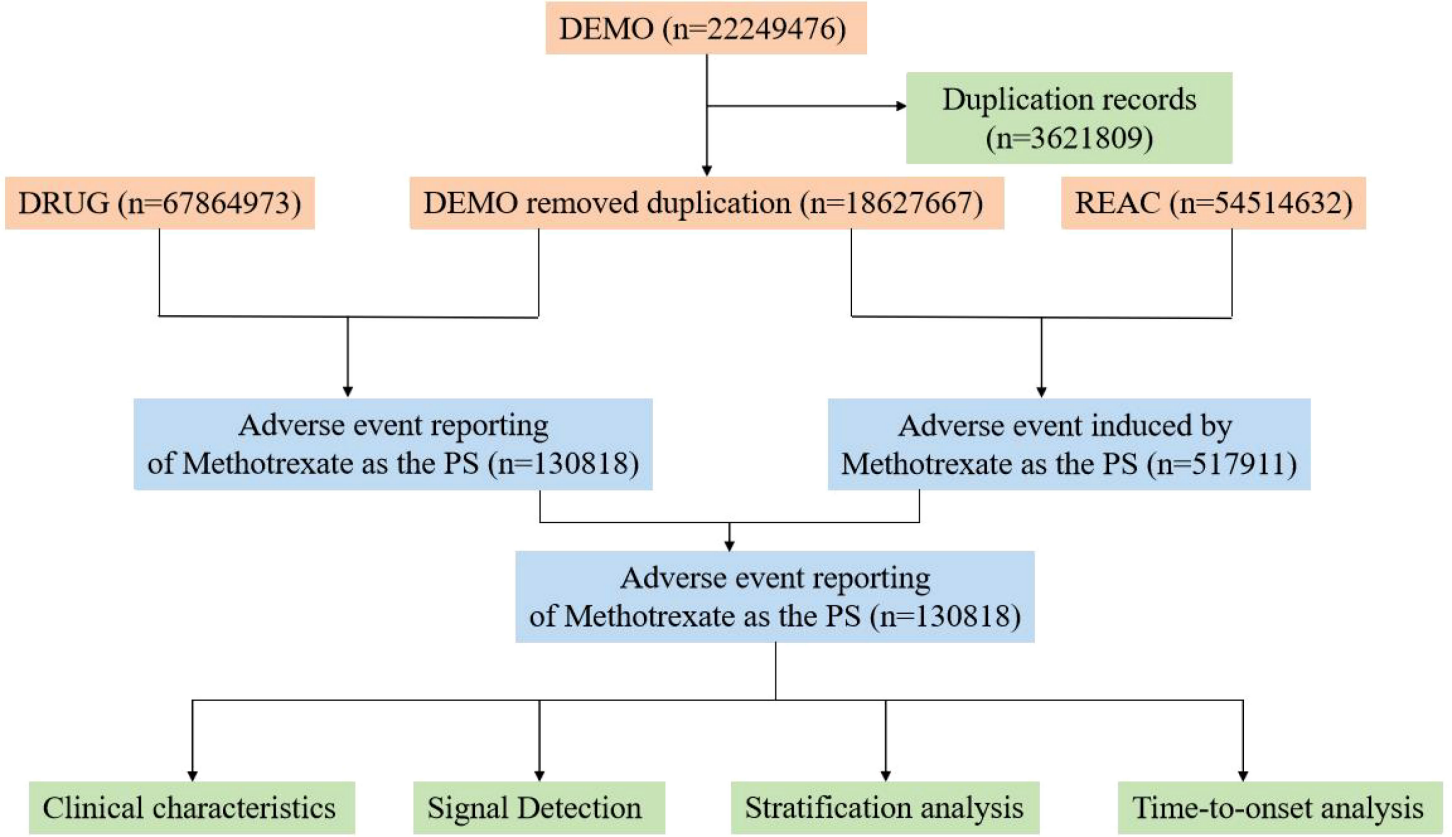
Flow-process diagram of MTX-related AEs from FAERS database.

This study compiled Adverse Event (AE) reports of the primary suspected (PS) drug from the FAERS database, retrieved between Q1–2004 and Q4 2024. To ensure specificity and accuracy, the search was limited to the generic name “MTX” and the brand name “Rheumatrex” as the primary suspected drugs. Data extraction and cleaning were performed using R version 4.4.2. The database includes 22,249,476 reports, which, after deduplication, resulted in 67,864,973 DRUG-related data reports. We extracted the PRIMARYID, CASEID, and FDADT fields from the DEMO table of the raw data and sorted them accordingly. For reports with the same CASEID, the report with the latest FDADT value was retained. If reports had the same CASEID and FDA_DT, the report with the largest PRIMARYID value was kept. Adverse events (AEs) were categorized according to the preferred terms (PT) and system organ classification (SOC) using the 27.0 version of the Medical Dictionary for Regulatory Activities (MedDRA) (15). **Figure 1** shows the flowchart of obtaining MTX-related events from the FAERS database.

### 2.2 Data analysis

We chose the Reporting Odds Ratio (ROR) as the primary signal detection method and used the proportion imbalance measurement method in the fourfold table to report adverse drug reaction data for MTX compared with other drugs (**Supplementary Table 1**). This study utilized several discriminant analysis methods, and the Reporting Odds Ratio (ROR) demonstrated strong effectiveness in early signal detection (16, 17). Methods such as ROR and Proportional Reporting Ratio (PRR) are widely used for detecting adverse drug reaction signals (18). Additionally, Bayesian Confidence Propagation Neural Network (BCPNN) methods (19) and Multi-item Gamma Poisson Shrinker (MGPS) methods (20) were also employed. Even in cases of fewer adverse drug reaction reports, MGPS and BCPNN remain powerful signal detection tools. The four statistical methods, ROR, PRR, BCPNN, and MGPS, were used in conjunction with all four thresholds to identify positive signals that met the criteria (**Supplementary Table 2**).

Additionally, to identify gender-based disproportional signals following MTX administration, the ROR method formula was used. Based on a 2x2 contingency table, we calculated the p-value using the Chi-square (χ²) test. Using the R package “ggplot2” (version 4.4.2) (21), a volcano plot was created, with the log2-transformed ROR values shown on the x-axis and the -log10-transformed adjusted p-values (P.adj, adjusted by FDR) on the y-axis. When ROR &gt; 1 and P.adj &gt; 0.05, it suggests that female patients are more likely to report a specific AE than male patients. Conversely, when ROR &lt; 1 and P.adj &lt; 0.05, it indicates that male patients are more likely to report a specific AE than female patients.

## 3 Results

### 3.1 Adverse events and population characteristics

A total of 130,818 AE reports associated with MTX were included in this study. **Table 1** presents baseline data for AE reports related to MTX. A significantly higher proportion of reports were from females (83,928, 64.2%) compared to males (38,348, 29.3%). In the reporting population for adverse reactions, adults aged 18-64.9 years (55,577, 42.5%) were the largest group, followed by those aged 65–85 years (31,324, 23.9%). Consistent with previous statements, the primary indication for MTX was rheumatoid arthritis (RA) (40,615, 31.0%), with acute lymphocytic leukemia (4,398, 3.4%) as a secondary indication. According to the FAERS database, the most frequently reported severe outcome was “Other Therapeutic” (OT) (52,686, 40.3%), followed by “Hospitalized” (HO) and “Death” (DE). The United States (46,017, 35.2%) led in the number of AE reports, followed by Canada (33,374, 25.5%) and Germany (9,244, 7.1%). The majority of the reporting population (45,326, 34.6%) was from the MD (medical doctor) category. **Supplementary Figure 1** shows the number of reported cases each year from the establishment of the FDA to the fourth quarter of 2024. The number of reports related to adverse reactions was the highest in 2019 (18,600 cases).Among the concomitant drugs (**Supplementary Table 4**), the drugs with the highest occurrence frequency were Prednisone(n=19763), Enbrel(n= 17,657), and Humira(n= 15,194).

**Table 1:** Basic information table.

ID	Overall
	(N=130818)
SEX	
F	83928 (64.2%)
M	38348 (29.3%)
Missing	8542 (6.5%)
WT	
<50 kg	3270 (2.5%)
>100 kg	2656 (2.0%)
50~100 kg	14940 (11.4%)
Missing	109952 (84.0%)
AGE	
<18y	9751 (7.5%)
>85y	1247 (1.0%)
18~64.9y	55577 (42.5%)
65~85y	31324 (23.9%)
Missing	32919 (25.2%)
OCCP_COD	
CN	20258 (15.5%)
HP	28905 (22.1%)
LW	115 (0.1%)
MD	45326 (34.6%)
OT	26951 (20.6%)
PH	5341 (4.1%)
RN	12 (0.0%)
Missing	3910 (3.0%)
REPORTER_COUNTRY	
UNITED STATES	46017 (35.2%)
CANADA	33374 (25.5%)
GERMANY	9244 (7.1%)

### 3.2 Signals associated with SOC levels

**Figure 2:**
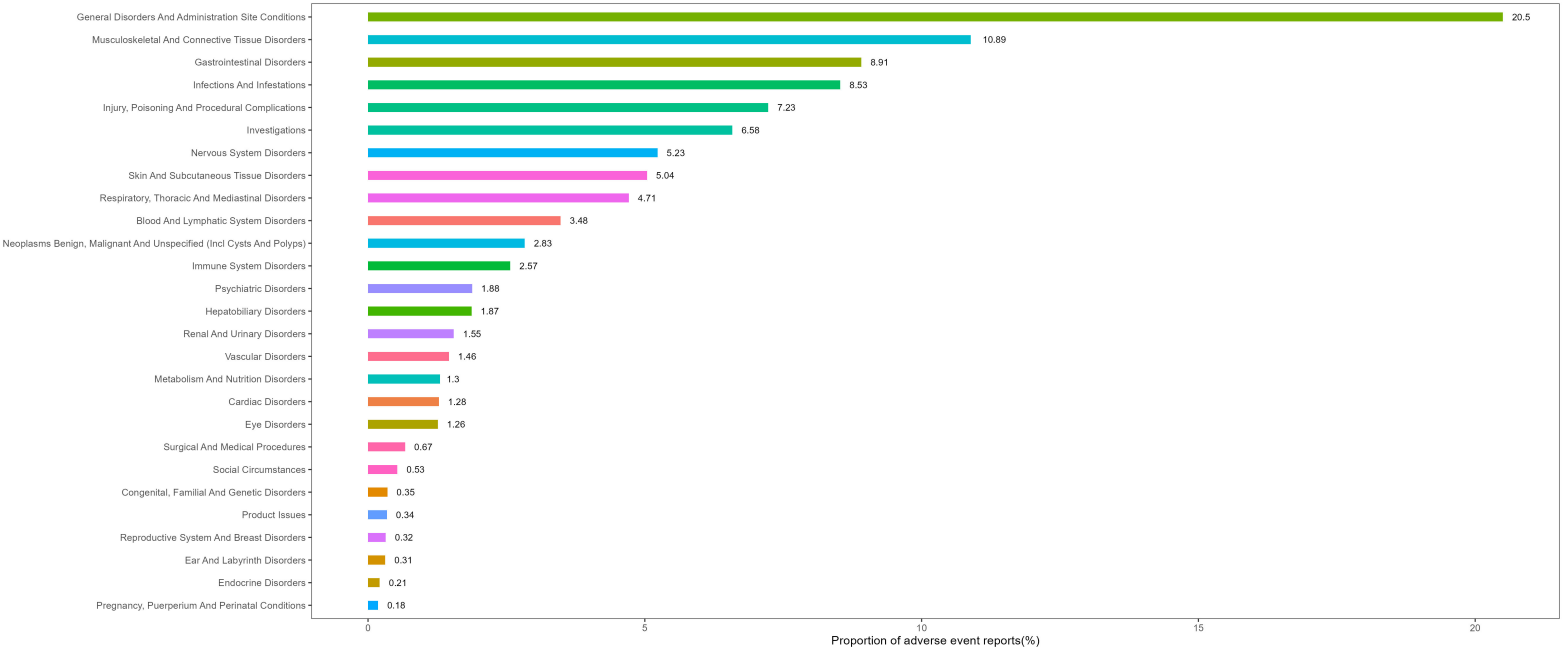
SOC ranking based on number of reports.

**Figure 3:**
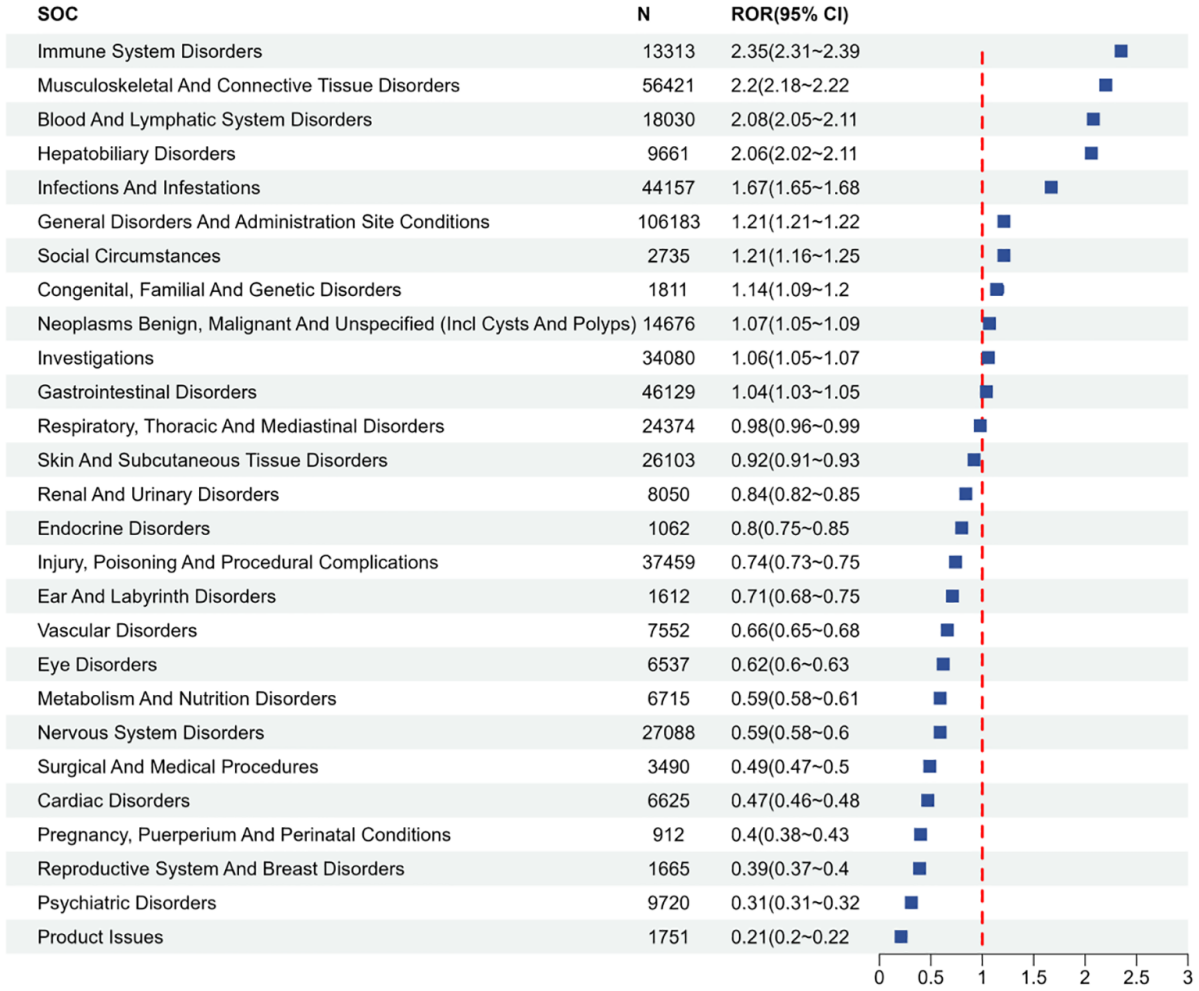
Lists the SOCs according to the top 27 confidence levels in the ROR.

**Figure 2** lists the proportions of 27 System Organ Classes (SOCs) related to MTX. **Figure 3** shows the signal strengths of MTX across various SOC levels in the FAERS database. The most commonly reported category was General Disorders and Administration Site Conditions [n = 106,183, ROR (95% CI) = 1.21 (1.21-1.22)], while the strongest signal was found in Immune System Disorders [n = 13,313, ROR (95% CI) = 2.35 (2.31-2.39)]. Several other SOCs demonstrated robust signal detection, including Musculoskeletal and Connective Tissue Disorders [n = 56,421, ROR (95% CI) = 2.20 (2.18-2.22)], Blood and Lymphatic System Disorders [n = 18,030, ROR (95% CI) = 2.08 (2.05-2.11)], Hepatobiliary Disorders [n = 9,661, ROR (95% CI) = 2.06 (2.02-2.11)], and Infections and Infestations [n = 44,157, ROR (95% CI) = 1.67 (1.65-1.68)]. Lack of statistical significance was observed when the lower limit of the ROR (95% CI) was below 1, for example, in categories such as Respiratory, Thoracic, and Mediastinal Disorders, Skin and Subcutaneous Tissue Disorders, and Renal and Urinary Disorders.

### 3.3 Signal detection at the PT level

**Figure 4:**
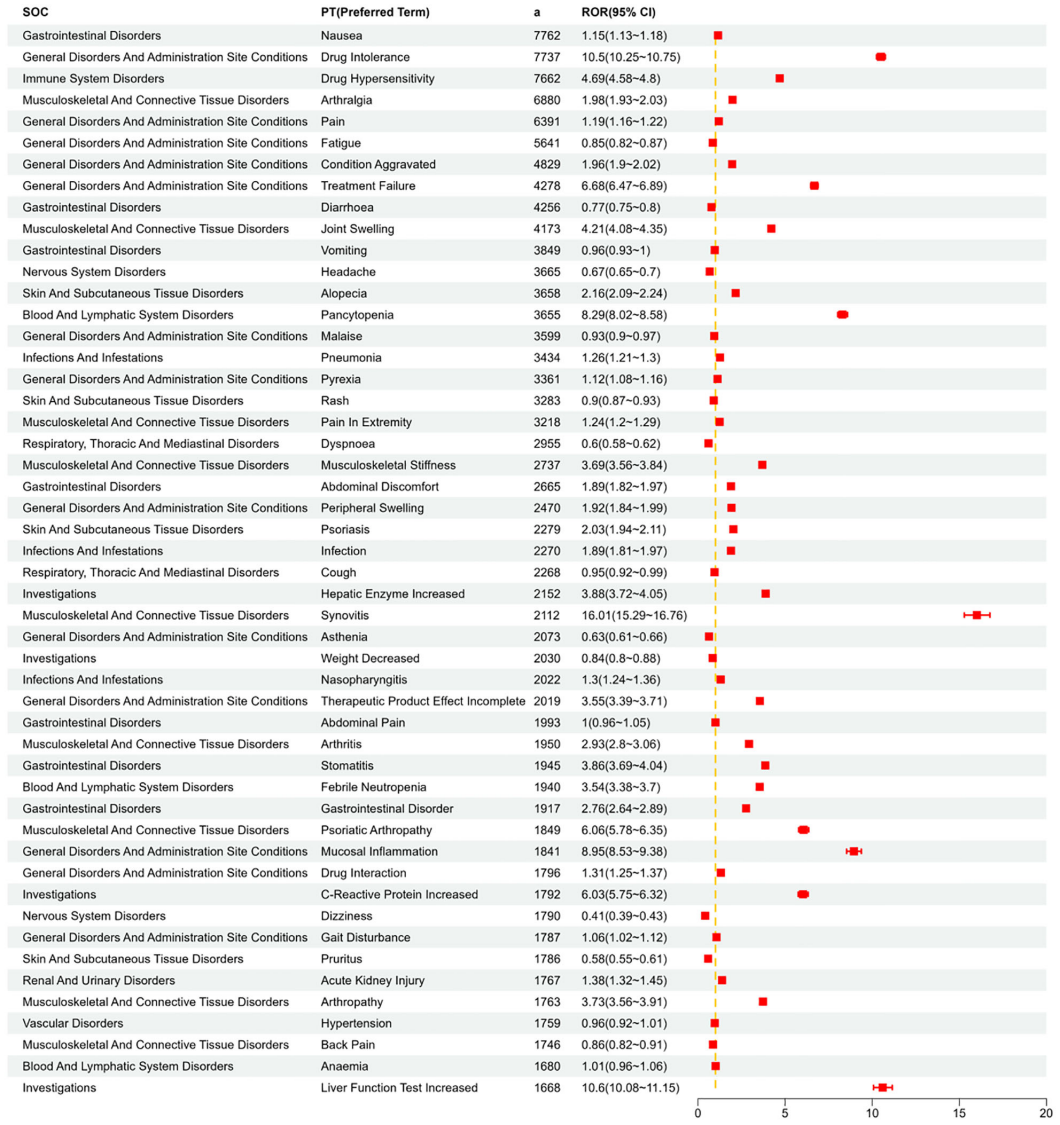
Visualizing the occurrence of the top 50 PT.

**Figure 5:**
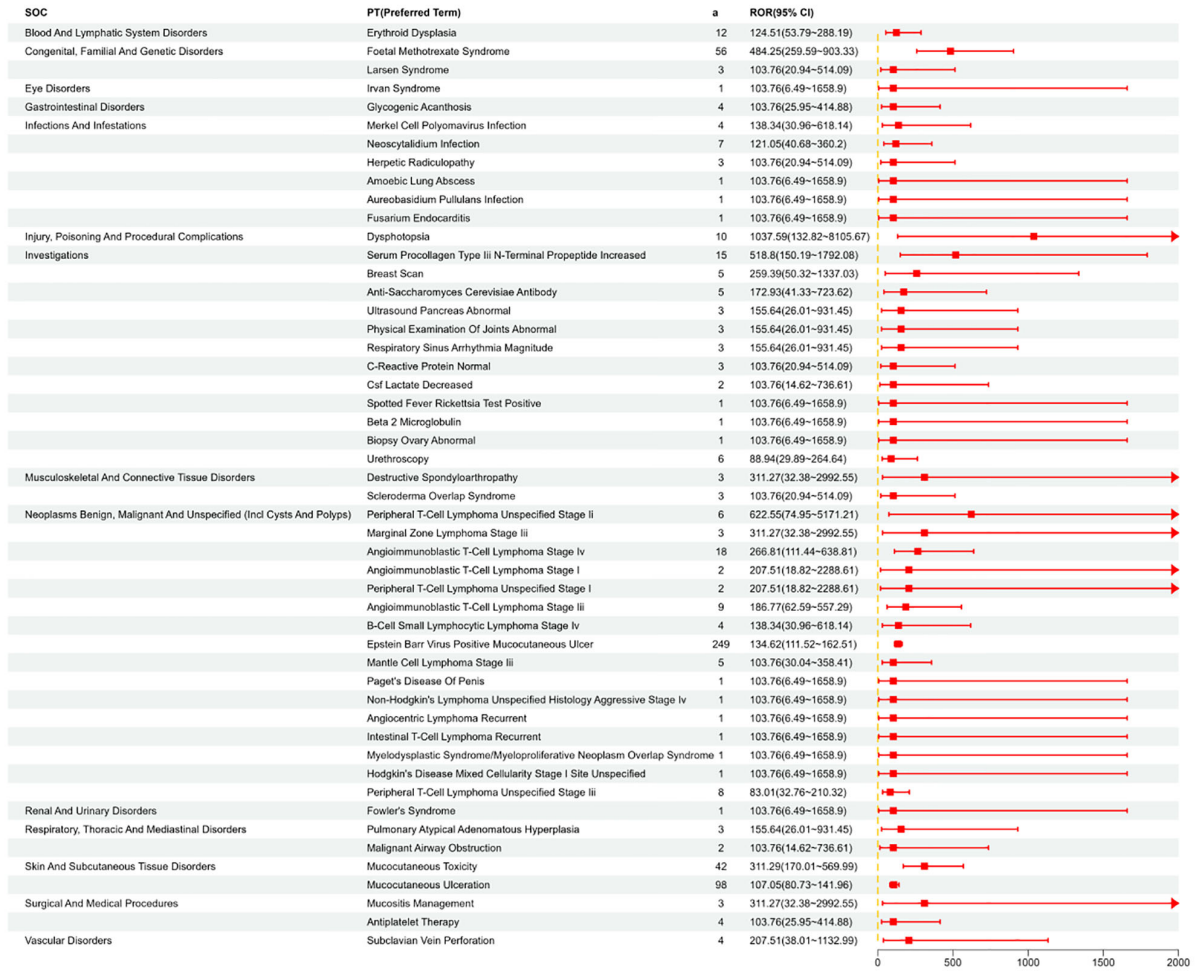
Shows the distribution of signal values for the first 50 PT according to the ROR (95%CI) method.

**Figure 6:**
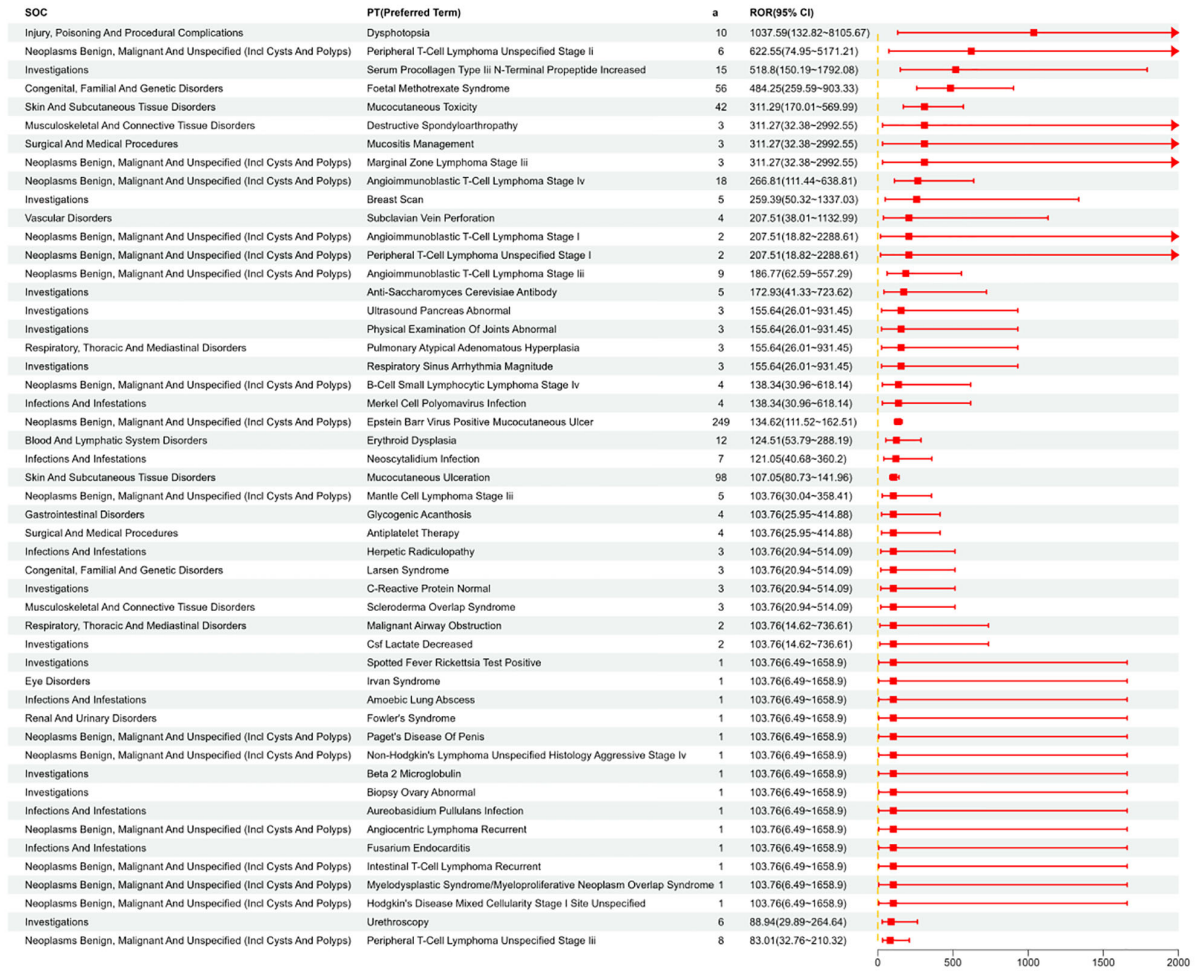
PT that meets ROR, PRR, BCPNN, and MGPS are all positive, and can be summarized according to SOC.

We systematically categorized and ranked all AEs related to MTX by report frequency. **Figure 4** displays the number of reports for different Preferred Terms (PTs) related to MTX in the FAERS database (top 50). **Figure 5** shows the signal strength of these PTs along with their corresponding SOCs (**Figure 6**). The top 10 most frequently occurring PT include Nausea, Drug Intolerance, Drug Hypersensitivity, Arthralgia, Pain, Fatigue, Condition Aggravated, Treatment Failure, Diarrhea, Joint Swelling. Additionally, all AEs that met the positive signal criteria and their corresponding SOCs are documented in **Supplementary Table 3**. Some PTs listed in the drug labeling include Nausea, Vomiting, Diarrhea, Oral Ulcers, Anorexia, Anemia, Leukopenia, Immune System Dysfunction, Elevated Liver Enzymes, Pneumonia, and Pulmonary Fibrosis. Potential adverse reactions not mentioned in the drug prescription information include Drug Allergy, Fatigue, Headache, Alopecia, etc.

### 3.4 Adverse event onset time

**Figure 7:**
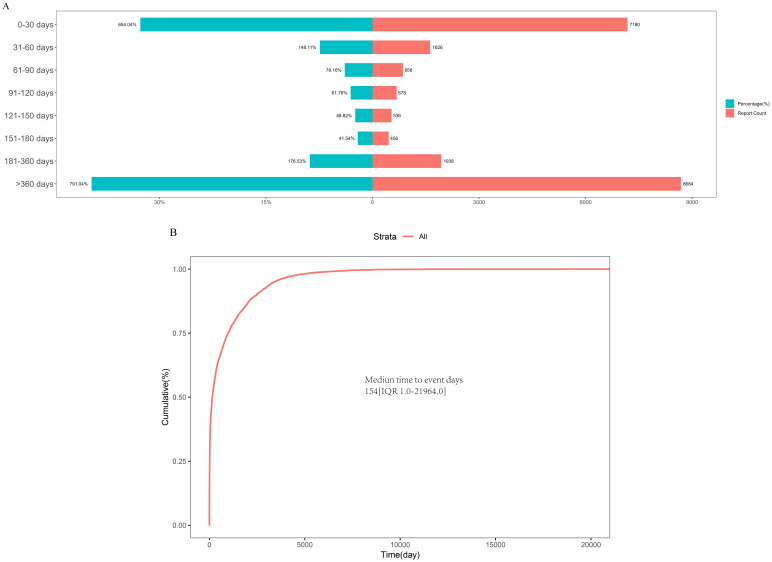
**(A)** Time to induce adverse reactions. **(B)** Adverse reaction induction time survival curve.

Regarding the occurrence time of AEs, we collected 21,956 reports documenting the time of AE onset (**Figure 7A**). Our analysis revealed that 36.56% (n = 8,684) of AEs occurred more than 360 days after MTX treatment. AEs were also observed within the following time frames: within 30 days (32.7%, n = 7,180), 31–60 days (7.4%, n = 1,626), 61–90 days (3.9%, n = 858), 91–120 days (3.01%, n = 678), 121–150 days (2.4%, n = 536), 151–180 days (2.1%, n = 456), and 181–360 days (8.8%, n = 1,938). As shown in **Figure 7B**, the median onset time for MTX-related AEs was 154 days, with an interquartile range (IQR) from 1 to 21,964 days.

### 3.5 PT analysis in subgroups

**Figure 8:**
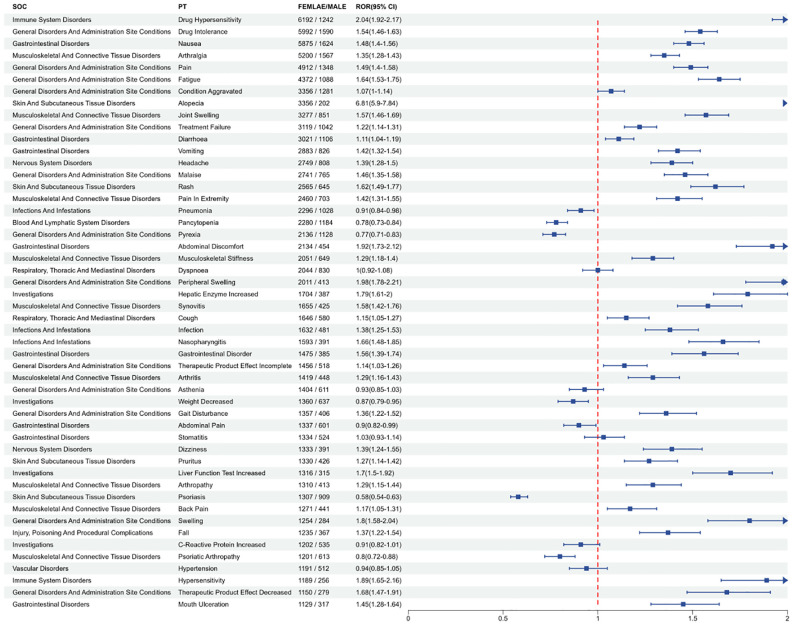
The proportion of male to female occurrence in the top 50 patients with PT was observed using ROR (95%CI) method.

**Figure 9:**
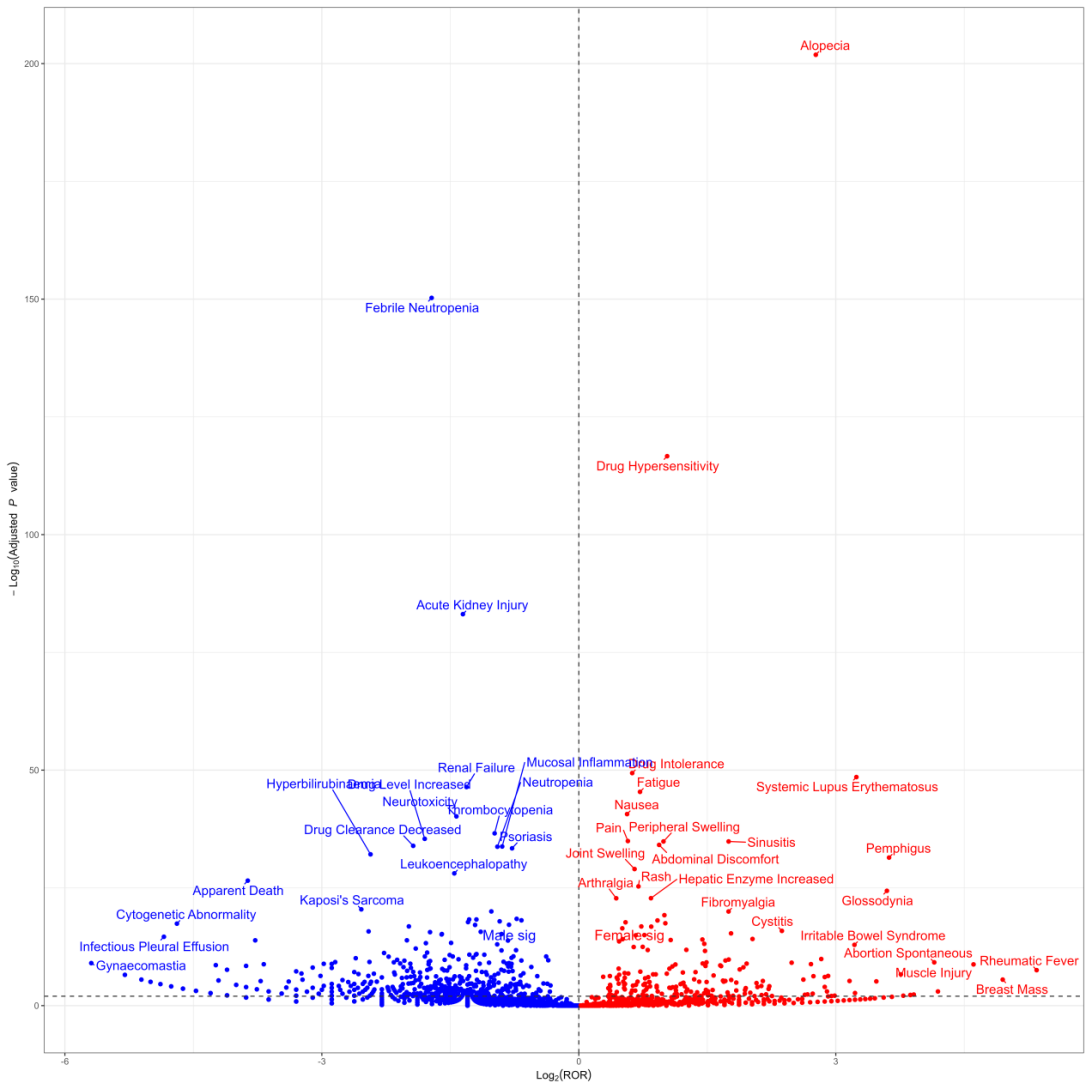
Sex difference risk signal volcano map.P.adj, the p-value is adjusted with false discovery rate(FDR) method.Red points indicate potential adverse events in female patients, while blue points denote those in male patients.

In the gender subgroup, as shown in **Supplementary Figure 2**, females were more likely to report Nausea and Drug Intolerance, while males more frequently reported Drug Hypersensitivity and Drug Intolerance. **Figure 8** shows the results of signal detection analysis at the PT level for males and females. Males were found to have a higher risk of PTs including Pneumonia, Pancytopenia, Pyrexia, Asthenia, Weight Decreased, Psoriasis, C-Reactive Protein Increased, Psoriatic Arthropathy, Hypertension, and Abdominal Pain. To further examine the gender differences in the adverse event signals detected for MTX, a visual representation using a volcano plot (**Figure 9**) was created, showing significant adverse event signals based on Log ^2^ ROR and -Log 10 p-values. As shown in **Supplementary Figure 3**, in the age subgroup, individuals under 18 years old were more likely to show Febrile Neutropenia, adults aged 18 to 65 most frequently reported Nausea, and those over 65 often experienced Drug Hypersensitivity. As shown in **Supplementary Figure 4**, among the reported population, consumers tend to be more aware of Drug Ineffective and Pain, while medical staff also report Drug Ineffective and Drug Hypersensitivity more frequently.

### 3.6 Onset time analysis in subgroups

In the gender subgroup, as shown in **Supplementary Figure 5**, the median onset time for females was 227 days, with an IQR from 1 to 21,964 days; for males, the median onset time was 78 days, with an IQR from 1 to 13,739 days. **Supplementary Figure 6** shows that in the age subgroup, the median onset time for individuals under 18 years old was 15 days, with an IQR from 1 to 5,569 days; for those aged 18–65 years, the median onset time was 104 days, with an IQR from 1 to 10,341 days; and for individuals over 65 years old, the median onset time was 296 days, with an IQR from 1 to 21,964 days. As shown in **Supplementary Figure 7**, in the reporting population, the median onset time for consumers was 28 days, with an IQR from 1 to 9,162 days; for healthcare professionals, the median onset time was 226 days, with an IQR from 1 to 21,964 days. The time-to-onset (TTO) of MTX-induced adverse drug events (ADEs) was defined as the interval between EVENTDT (occurrence time in the DEMO table) and STARTDT (treatment start time in the THER file).

## 4 Discussion

This study provides a comprehensive analysis of adverse event (AE) reports related to MTX, collected through the FAERS database from Q1–2004 to Q4 2024. The findings reveal the safety profile of MTX as an immunomodulator in clinical use and offer deeper insights into its adverse reactions.

### 4.1 Overall distribution of adverse events

Using various signal detection methods, we identified potential safety concerns associated with MTX use, particularly highlighting differences across gender and age subgroups. We observed a substantial number of MTX-related AE reports, with a total of 130,818 cases recorded, indicating a broad range of adverse reactions that MTX may induce in clinical use. These AEs predominantly affected the immune system [n=13,313, ROR (95% CI)=2.35 (2.31–2.39)], musculoskeletal system [n=56,421, ROR (95% CI)=2.2 (2.18–2.22)], and hematologic system [n=18,030, ROR (95% CI)=2.08 (2.05–2.11)], with the occurrence of different adverse reactions significantly associated with patients’ gender and age. For instance, female patients reported a significantly higher number of AEs than males, possibly due to the wider use of MTX in females and differential immune system responses. Notably, the most commonly reported system organ class was “General Disorders And Administration Site Conditions” [n=75,761, ROR (95% CI)=1.26 (1.25–1.27)], while the category with the strongest signal was “Immune System Disorders” [n=9,839, ROR (95% CI)=2.39 (2.34–2.44)]. These findings align with MTX’s known mechanism of action, where its immunosuppressive properties may be the underlying cause of immune system disorders.

Signal detection conducted through multiple statistical methods (including ROR, PRR, BCPNN, and MGPS) further validated the risks of MTX-related AEs. These methods provided positive signals at different thresholds, offering evidence for the safe clinical use of MTX. In particular, we found that the signal strength for the “Immune System Disorders” category was especially prominent [ROR (95% CI)=2.35], consistent with MTX’s characteristic of triggering immune system-related adverse reactions through immune suppression. Additionally, a strong signal was observed in the “Blood And Lymphatic System Disorders” category [ROR (95% CI)=2.08 (2.05–2.11)], which may be related to MTX-induced bone marrow suppression and hematologic toxicity.

#### 4.1.1 Time correlation of SOC related to MTX

We categorized the occurrence times and months of methotrexate-related SOCs (**Supplementary Table 6**) and found the following: Renal and urinary disorders (median_TIME 12 days), Skin and subcutaneous tissue disorders (median_TIME 27 days), Hepatobiliary disorders (median_TIME 32 days), musculoskeletal and connective tissues disorders (median_TIME 591 days), surgical and medical procedures (median_TIME 795 days), and Neoplasms, Benign, Malignant and Unspecified (median_TIME 1096 days).Renal and urinary disorders had a median onset time of 12 days, indicating a relatively early onset of adverse reactions. This could be related to the direct toxic effect of methotrexate on the renal tubules. Common symptoms include hematuria, proteinuria, and abnormal kidney function. Methotrexate’s renal toxicity is closely related to dose and treatment duration, and long-term use may lead to chronic kidney damage, particularly in patients with pre-existing renal impairment, who are at higher risk. Therefore, it is recommended to regularly monitor kidney function during treatment. Skin and subcutaneous tissue disorders had a median onset time of 27 days, representing early adverse reactions. Common symptoms include rash, itching, hair loss, and increased photosensitivity. Skin reactions are relatively common during methotrexate treatment, especially in patients sensitive to sunlight. Mild reactions typically resolve after dose reduction or symptomatic treatment, but severe reactions such as Stevens-Johnson syndrome, though rare, should be closely monitored. Hepatobiliary disorders had a median onset time of 32 days, representing early to mid-term adverse reactions. Common symptoms include abnormal liver function, jaundice, and fatty liver. Hepatotoxicity is one of the common adverse reactions of methotrexate. Long-term use may lead to liver fibrosis and cirrhosis. Studies suggest regular liver function monitoring during treatment and avoiding concurrent use with other hepatotoxic drugs.”

The median time for adverse reactions related to the musculoskeletal and connective tissues is 591 days, which falls under long-term adverse effects. Common symptoms include joint pain, bone marrow suppression, and muscle weakness. Long-term use of methotrexate may lead to osteoporosis and joint damage. It is recommended to supplement with calcium and vitamin D during treatment, and use bone protectors if necessary. The median time for adverse reactions related to surgical and medical procedures is 795 days, which falls under very late-stage adverse effects. This may be related to complications of treatment requiring surgical intervention. Long-term use of methotrexate may increase the risk of certain surgical complications, such as infection and difficulty with postoperative recovery. Therefore, the surgical risk should be comprehensively evaluated during treatment. The median time for Neoplasms, Benign, Malignant and Unspecified is 1096 days, which falls under very late-stage adverse effects. This may be related to the long-term use of methotrexate, especially for malignancies such as hepatocellular carcinoma and non-Hodgkin lymphoma, particularly for patients with a history of such conditions. Tumor markers should be closely monitored during treatment.

#### 4.1.2 Seasonal correlation of SOCs related to methotrexate

In terms of SOC and frequency (**Supplementary Table 7**):Gastrointestinal Disorders: These showed higher frequencies across all quarters, especially in Q2 and Q4 (896 and 919), indicating that these adverse reactions may be related to seasonal changes or variations in medication usage. General Disorders and Administration Site Conditions: These had relatively consistent high frequencies across all quarters, with Q1’s 973 being the highest for the year. However, other quarters also exhibited relatively high frequencies, which may be related to the method of drug administration and common issues during the treatment process. Infections and Infestations: Q1 (1080) and Q4 (1047) exhibited particularly high frequencies, suggesting that infection and parasite issues might be closely related to seasonal changes or the timing of treatment. Congenital, Familial, and Genetic Disorders: Frequencies were relatively low across all quarters, generally ranging between 10 and 30, indicating that the occurrence of these adverse reactions may be less frequent or more individual. Social Circumstances: The frequency of these events was also relatively low, with minimal variation across the quarters, which may be due to the limited impact the medication itself has on social environment factors. SOC and Seasonal Aspects: Gastrointestinal Disorders and Infections and Infestations showed some seasonal fluctuations. For example, Infections and Infestations peaked in Q1 and Q4, which may be linked to seasonal transitions or climate changes. Q1 is typically flu season, which could lead to an increase in infection-related symptoms, while Q4 may be associated with colder weather and winter flu. Nervous System Disorders: These peaked in Q2 (719), which could be related to weather changes, seasonal influences, or variations in the duration of medication use. Nervous system issues may have different triggers depending on the season. Certain quarters may see fluctuations in medication usage, particularly during specific periods or seasons (e.g., flu season), which may lead to an increase in the frequency of certain adverse reactions. For example, the high frequency of Infections and Infestations in Q1 may be due to the winter flu season or a greater impact on the immune system from the medication. Changes in weather or peaks in viral infections (such as in winter) may increase the occurrence of some conditions like Gastrointestinal Disorders and Infections and Infestations. Patient Population Characteristics: Certain conditions, such as Congenital, Familial, and Genetic Disorders, have a more fixed incidence rate and are less affected by seasonal or dosage variations, resulting in lower frequencies. In different quarters, changes in treatment plans or medication adjustments may influence the occurrence of various adverse reactions. For instance, Gastrointestinal Disorders and Musculoskeletal and Connective Tissue Disorders exhibited higher frequencies in Q1 and Q4, possibly due to the patient’s treatment progress or the duration of medication use.

#### 4.1.3 Correlation of adverse reactions of methotrexate with gender and age

This study’s analysis of gender and age subgroups revealed significant population differences in MTX-related adverse reactions (**Supplementary Figures 5, 6**). For instance, female patients typically experienced a longer time to onset of AEs when using MTX. This may be related to variations in the female immune system and hormonal levels. Females generally exhibit stronger immune responses, which, under MTX’s immunosuppressive effects, may lead to the gradual emergence of certain adverse reactions, such as nausea [ROR (95% CI)=1.08 (1.05–1.11)] and drug intolerance [ROR (95% CI)=10.89 (10.6–11.19)]. These reactions may manifest only after prolonged drug use. Additionally, fluctuations in hormone levels, such as estrogen, may influence MTX metabolism and immune responses, resulting in delayed AEs in females. Male patients, on the other hand, often reported more severe AEs, such as drug hypersensitivity [ROR (95% CI)=4.33 (4.09–4.58)] and pancytopenia [ROR (95% CI)=7.17 (6.76–7.61)].This may be because men exhibit different characteristics in their immune system responses, leading to stronger immune reactions and, consequently, more severe side effects. The immune system in male patients may respond more intensely to the immunosuppressive effects of methotrexate, which can lead to more serious immune-related adverse events.

In terms of age, elderly patients have a significantly increased risk of drug allergies and immune system-related AEs, which may be associated with the aging of the immune system and physiological factors such as reduced liver and kidney function. The immune system in older adults declines, and T cell function weakens, which can make the immune response to drugs less stable. As methotrexate is an immunosuppressive agent, it may cause more severe immunosuppressive effects in elderly patients with already weakened immune systems, thereby increasing the risk of immune-related AEs. Additionally, elderly patients’ metabolic capacity is reduced, and the half-life of methotrexate is prolonged, which may lead to the accumulation of the drug in the body, increasing toxicity and triggering more side effects. For patients under 18 years old, the onset time of AEs is shorter, which may be related to the physiological characteristics of adolescents and children. The immune system in younger patients may be more active than in adults, meaning they may respond more rapidly to the immunosuppressive effects of methotrexate. Furthermore, young individuals typically have better liver and kidney function, leading to faster drug metabolism. As a result, the drug may quickly reach certain concentrations in the body, leading to earlier onset of adverse reactions.

The differences in immune responses among patients of different genders and ages are the fundamental reasons for the occurrence of methotrexate-related AEs. Gender differences in drug metabolism in women may be related to fluctuations in hormone levels. Estrogen and other hormones may affect immune responses and the rate of drug metabolism, thus influencing the manifestation of adverse drug reactions. For example, estrogen’s role in immune response may enhance women’s reactions to immunosuppressive drugs, leading to delayed adverse reactions. The immune system in younger patients is more active and responsive, which may explain why they are more likely to experience adverse reactions to methotrexate in a shorter time, especially in terms of immune-related AEs.

#### 4.1.4 Concomitant drugs related to adverse reactions of methotrexate

In terms of concomitant medications(**Supplementary Table 4**), Prednisone is a corticosteroid with immunosuppressive and anti-inflammatory effects. It can enhance the immunosuppressive action of methotrexate, increasing the risk of infections. The cumulative immunosuppressive effect may lead to an increased risk of bacterial and viral infections. In addition, long-term use of corticosteroids may also increase the risk of osteoporosis. Enbrel is an anti-TNF-α drug, and when used in combination with methotrexate, it may enhance the immunosuppressive effects, increasing the risk of bacterial, fungal, and viral infections. Additionally, the risk of liver dysfunction and hematological abnormalities (such as anemia and leukopenia) may increase. Humira is another anti-TNF-α drug, similar to Enbrel. When used in combination with methotrexate, it may exacerbate the immunosuppressive effects, especially increasing the risk of infections such as tuberculosis and fungal infections, as well as liver toxicity and hematological adverse effects. Sulfasalazine and Leflunomide are used to treat inflammatory diseases. When used in combination with methotrexate, they may enhance the immunosuppressive effects, increasing the risk of hematological adverse effects (such as leukopenia and anemia). Gastrointestinal discomfort, skin rashes, and liver dysfunction may also occur. When methotrexate is used in combination with other medications, drug interactions may exacerbate immunosuppressive effects, increasing the risk of infections, liver damage, and hematological abnormalities. Through detailed clinical data analysis and pharmacological evaluation, these potential risks can be identified, providing valuable reference for clinical treatment.

#### 4.1.5 Fatal events related to methotrexate

In terms of fatal Preferred Terms (PTs), we observed that the top 10 most frequent PTs were Death (1097/2442), Pancytopenia (982/2442), Sepsis (656/2442), Toxicity To Various Agents (614/2442), Pneumonia (538/2442), Respiratory Failure (465/2442), Septic Shock (461/2442), Multiple Organ Dysfunction Syndrome (427/2442), Drug Ineffective (413/2442), and Acute Kidney Injury (327/2442) (see **Supplementary Table 5** for details). MTX’s fatal reactions are typically compounded by its adverse effects, particularly its toxic impact on multiple organs.

When used in high doses or improperly, the immunosuppressive effects of MTX may make patients more susceptible to infections, leading to conditions such as sepsis and septic shock. Bone marrow suppression results in pancytopenia, which further exacerbates immune system damage. Patients with hepatic diseases are more likely to experience organ toxicity when using MTX. Methotrexate is primarily metabolized in the liver, where it is converted into active metabolites. However, liver dysfunction can impair the metabolism of methotrexate, causing the drug to accumulate in the body and increasing the risk of toxic reactions. Methotrexate is mainly excreted through the kidneys, and therefore, patients with renal insufficiency often face limited excretion of the drug, leading to its accumulation and an increase in toxic reactions.

Although this study extracted a large number of MTX adverse reaction reports from the FAERS database, some potential and uncommon side effects remain underreported. For example, adverse reactions in categories such as Pregnancy, Puerperium, and Perinatal Conditions [ROR (95% CI)=0.4 (0.38–0.43)], Immune System Disorders [ROR (95% CI)=2.35 (2.31–2.39)], Infections and Infestations [ROR (95% CI)=1.67 (1.65–1.68)], Metabolism and Nutrition Disorders [ROR (95% CI)=0.59 (0.58–0.61)], Musculoskeletal and Connective Tissue Disorders [ROR (95% CI)=2.2 (2.18–2.22)], Psychiatric Disorders [ROR (95% CI)=not provided], and Vascular Disorders [ROR (95% CI)=0.31 (0.31–0.32)] were reported but not explicitly listed in the drug’s prescribing information. This suggests that the spectrum of MTX side effects may be broader than currently recognized, necessitating heightened clinical vigilance for these adverse reactions and further clinical validation.

### 4.2 Important SOC analysis related to MTX

MTX has been used for over 60 years and, in recent years, has been primarily applied in the treatment of autoimmune diseases such as rheumatoid arthritis (RA) and psoriatic arthritis, though it can also be used alone or in combination for other connective tissue diseases (22). However, MTX is toxic to all rapidly dividing cells (23). This toxicity ranges from mild gastrointestinal side effects to more severe hematologic, hepatic, pulmonary, and renal effects. Some studies suggest that its therapeutic efficacy and side effects are related to ethnicity (24). Our research found that the frequency of drug intolerance[n =7737,ROR(95%Cl)10.5 (10.25 - 10.75)] and Treatment Failure[n =4278,ROR(95%Cl)(6.68 (6.47 - 6.89)] was highest in the signal category “General Disorders and Administration Site Conditions,” which may be related to the fact that DMARDs typically show benefits only after about six months (25). Despite this, MTX has good efficacy and safety profiles (26), and remains the first-line treatment (27). Some studies have tested the tolerability and availability of high-dose self-administered SCMTX for five weeks in patients with RA or psoriatic arthritis (28). Additionally, 221 patients maintained low-dose MTX treatment for up to 23 years without experiencing intolerance, although this requires further clinical observation. Regarding gastrointestinal disorders, literature reports that gastrointestinal adverse effects [n =46129,ROR(95%Cl)1.04 (1.03-1.05)]are the most common side effects (29, 30). Our study showed that gastrointestinal adverse effects ranked third in frequency. These gastrointestinal adverse effects include nausea, vomiting, and abdominal discomfort (31), which generally align with the adverse effects we observed. These side effects are dose-dependent (32). These adverse reactions are common but directly impact the discontinuation of MTX treatment (33). The pathogenesis of gastrointestinal side effects involves multiple organs, with some studies showing a correlation with plasma homocysteine levels (34). Other studies have found an association with the SLC19A1 80G allele (35). Oral absorption may also increase the frequency of diarrhea in patients (26, 36). Current guidelines recommend taking folic acid the day after MTX administration to significantly reduce gastrointestinal side effects and thus lower the risk of non-compliance with treatment (37). Additional medications that can be taken include the proton pump inhibitor pantoprazole (38) to prevent peptic ulcers.

Respiratory, Thoracic, and Mediastinal Disorders: Studies have shown that approximately 2-7% of patients using MTX experience lung involvement, and among rheumatic disease patients using MTX, about 12.5%-33% develop acute pulmonary toxicity (39, 40). MTX-induced pulmonary involvement is caused by various factors, such as MTX’s interference with folate metabolism, hypersensitivity reactions to the drug, and immune suppression that makes patients more susceptible to infections (12). Literature also discusses the relationship between MTX pulmonary toxicity and dosage, with reports indicating that patients receiving only 12.5 mg of MTX as the total dose have experienced life-threatening acute pneumonia, which represents the lowest reported dose for pulmonary toxicity in RA patients (41, 42).

Hepatobiliary Disorders: In our study, elevated liver enzymes, increased alanine aminotransferase (ALT), and increased liver function tests (LFTs) can be categorized as the same symptom clinically. According to our incomplete statistics, the occurrence of Hepatobiliary Disorders[n=9661,ROR(95%Cl)2.06 (2.02 - 2.11)] only accounts for 1.87% of all Standard Occurrence Categories (SOC). However, research indicates that liver toxicity caused by MTX is second in frequency to gastrointestinal adverse effects (43). Within the first three years of MTX use in RA patients, the incidence of elevated liver enzymes is 13%, with a cumulative incidence of 31% (43). The exact cause of MTX-induced liver toxicity remains unclear, but some studies suggest that MTX-polyglutamate (MTX-PG), a metabolite of MTX, accumulates in hepatocytes and triggers oxidative stress, inflammation, fatty degeneration, fibrosis, and cell apoptosis, leading to liver oxidative stress (44). Non-alcoholic fatty liver disease (NAFLD) might be a potential condition contributing to persistent transaminase elevation in RA patients undergoing MTX treatment (45). The use of different dosages and combinations with various anti-rheumatic drugs is also a contributing factor (46).

Therefore, during MTX treatment, regular monitoring of liver damage in patients is particularly important. Studies have confirmed (47) that in patients with a history of liver disease or those who experience early and mild liver damage caused by MTX, liver biopsy is more reliable and useful than any other clinical examination, especially in cases where liver damage has multiple etiologies beyond MTX. As for improving liver damage, one study observed (48) that after reducing the dose or discontinuing MTX, liver enzymes returned to normal levels, indicating that appropriate dose control of MTX is an important strategy for reducing liver damage.

In terms of Nervous System Disorders: MTX is not only a medication for treating rheumatic autoimmune diseases but also a key drug for treating various cancers in both children and adults (49). However, MTX can cause severe neurotoxicity, which is a side effect that increases the risk of death (50). Since the 1970s, researchers have found that MTX is associated with disseminated necrotizing leukoencephalopathy and other neurotoxic sequelae. The exact mechanism of MTX-induced neurotoxicity has yet to be established (51). MTX neurotoxicity can present as seizures, focal neurological deficits, or diffuse encephalopathy. Stroke-like symptoms (52, 53), reversible posterior leukoencephalopathy syndrome (54), and rare complications such as myelopathy and polyneuropathy (55, 56) are also symptoms of MTX neurotoxicity. As for prevention and treatment, there is currently no definitive remedy for MTX-induced neurotoxicity. For example, steroids are only used to alleviate vasogenic edema, and combination therapy with folinic acid (57), metformin (58), and theophylline (59) lacks conclusive evidence. This requires clinicians to possess the ability to predict and recognize these various manifestations, as well as to understand the side effects of MTX in different populations.

In terms of Cardiac Disorders: Our study found that pericarditis was the most common condition among cardiac disorders[n=313,ROR(95%Cl),2.42(2.17-2.71)], which contrasts with the literature reporting that pericarditis is a rare complication of MTX. The first case of pericarditis as a complication of MTX was reported in a pregnant woman undergoing treatment for a hydatidiform mole (60). Research has shown that the use of MTX in early pregnancy increases the risk of congenital heart disease in newborns (61). This is because MTX inhibits DHFR, which is a key enzyme involved in nucleotide synthesis and methylation, both of which play important roles in embryonic development. Another study (62) found that in the MTX treatment group, embryos exhibited dilation or underdevelopment of the ventricles and atria, delayed heart development, abnormal heart twisting, and impaired ventricular contractility. Although the FDA’s black box warning does not mention heart disease, our study and the literature on MTX complications suggest that, although such complications are rare, clinicians should remain vigilant when using MTX.

## 5 Limitations

Although this study conducted a comprehensive analysis of MTX’s adverse reactions using the FAERS database, providing valuable insights for clinical medication use, several limitations remain. 1.Data Source and Reporting Bias: The FAERS database is a spontaneous reporting system, which has inherent issues with underreporting and reporting bias. Some adverse reactions may not be reported for various reasons, such as patients being unaware or healthcare providers failing to recognize them, which may lead to an underestimation of the true incidence of adverse events. Moreover, the varying backgrounds and levels of understanding among reporters can lead to discrepancies in the accuracy and completeness of reports. For example, differences in the focus of consumers and healthcare professionals may cause biases in the reporting of certain adverse reactions, failing to provide a comprehensive representation of MTX’s adverse effects. 2.Limited Causal Inference: The data in the FAERS database primarily provide associative information, making it challenging to establish a clear causal relationship between MTX and adverse reactions. Although various statistical methods are used to detect signals, other factors that may influence the occurrence of adverse reactions, such as underlying conditions and concomitant medications, may not be comprehensively recorded in the database. This lack of detailed information could interfere with the accurate assessment of MTX’s adverse effects. 3.Lack of Dosage and Treatment Details: Critical information regarding MTX dosage, treatment duration, and other key parameters may be missing or inaccurate in the database, hindering the ability to conduct in-depth analysis of the dose-response relationship. For instance, when studying hepatotoxicity, different dosages and the use of multiple anti-rheumatic drugs are known to be contributing factors. However, due to the absence of detailed dosage data, it is difficult to accurately assess how dosage influences the occurrence of adverse reactions, and to define the safe and effective dosage range. 4.Subgroup Analysis Limitations: In subgroup analyses based on factors such as gender and age, there may be an unequal distribution of sample sizes, which could affect the generalizability and reliability of the results. For example, variations in sample size across different age groups might lead to biased estimates of the incidence of certain adverse reactions in specific age groups, making it difficult to accurately reflect the true risks across various age groups.

## 6 Conclusion

This study analyzed a large number of adverse event reports related to MTX, revealing its safety profile. Adverse reactions were found to affect multiple systems, with observed differences across genders and ages, and new potential adverse reactions were identified. However, due to the study’s limitations, clinical use of the drug still requires monitoring based on individual patient conditions, and further research is necessary to validate its full spectrum of side effects.
