# Supplementary material for: Adverse drug reactions related to methotrexate: a real-world pharmacovigilance study using the FAERS database from 2004 to 2024
Source: Front Immunol. 2025 Jun 4;16:1586361. doi: 10.3389/fimmu.2025.1586361 (PMC12174053; doi:10.3389/fimmu.2025.1586361)
Supplement: Supplementary file 1 [file DataSheet1.docx]

Supplementary Figure 1. Annual report chart


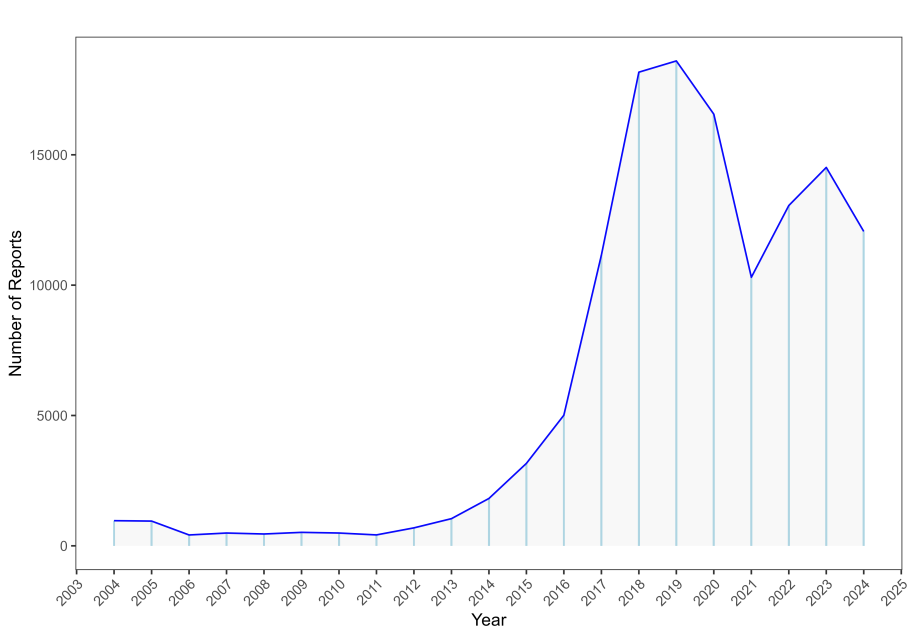


Supplementary Figure 2. Listing the top 50 PT according to the ROR method and analyzing the association with gender.


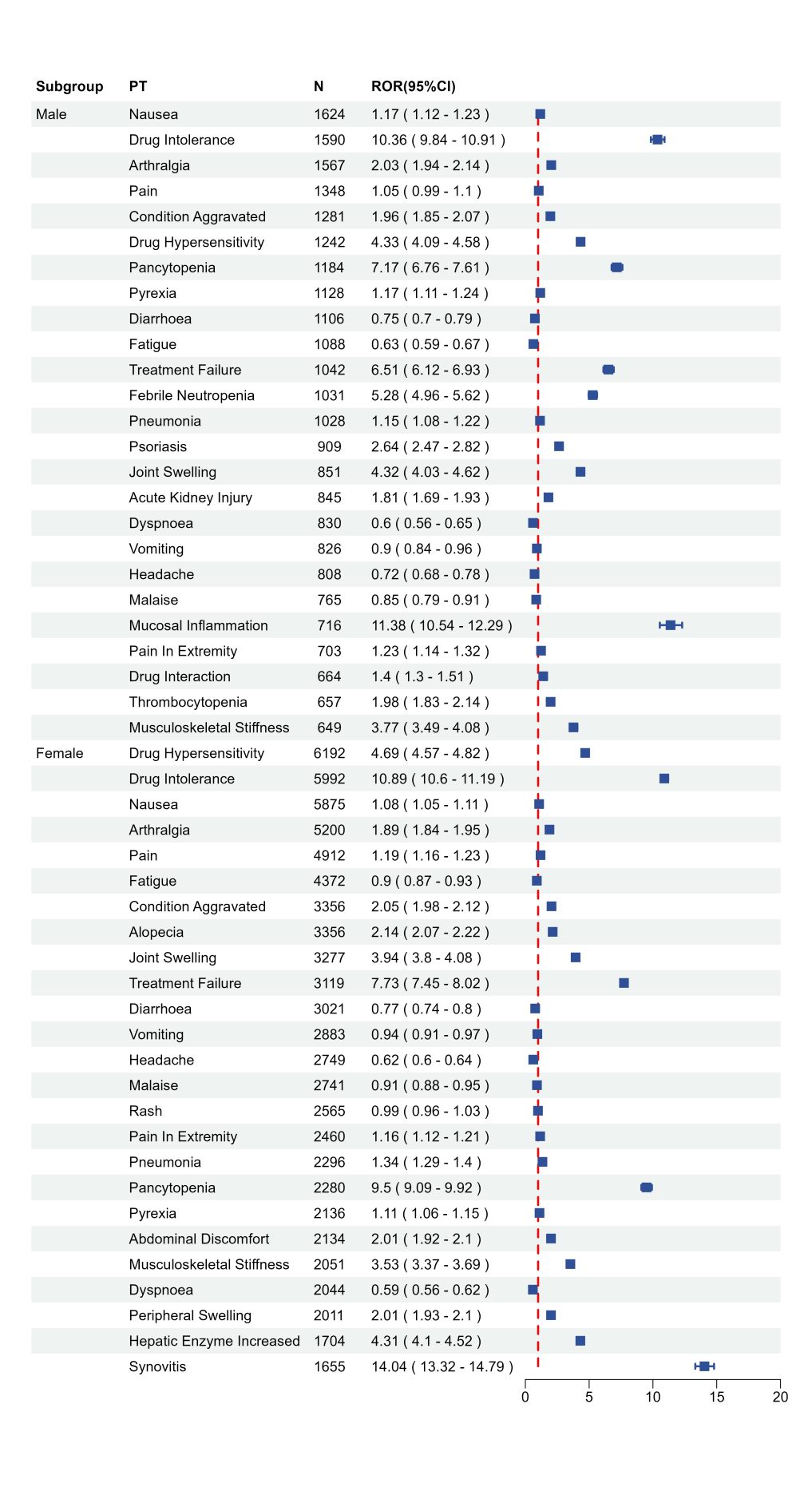


Supplementary Figure 3.PT signal strength of the top 15 in different age groups.


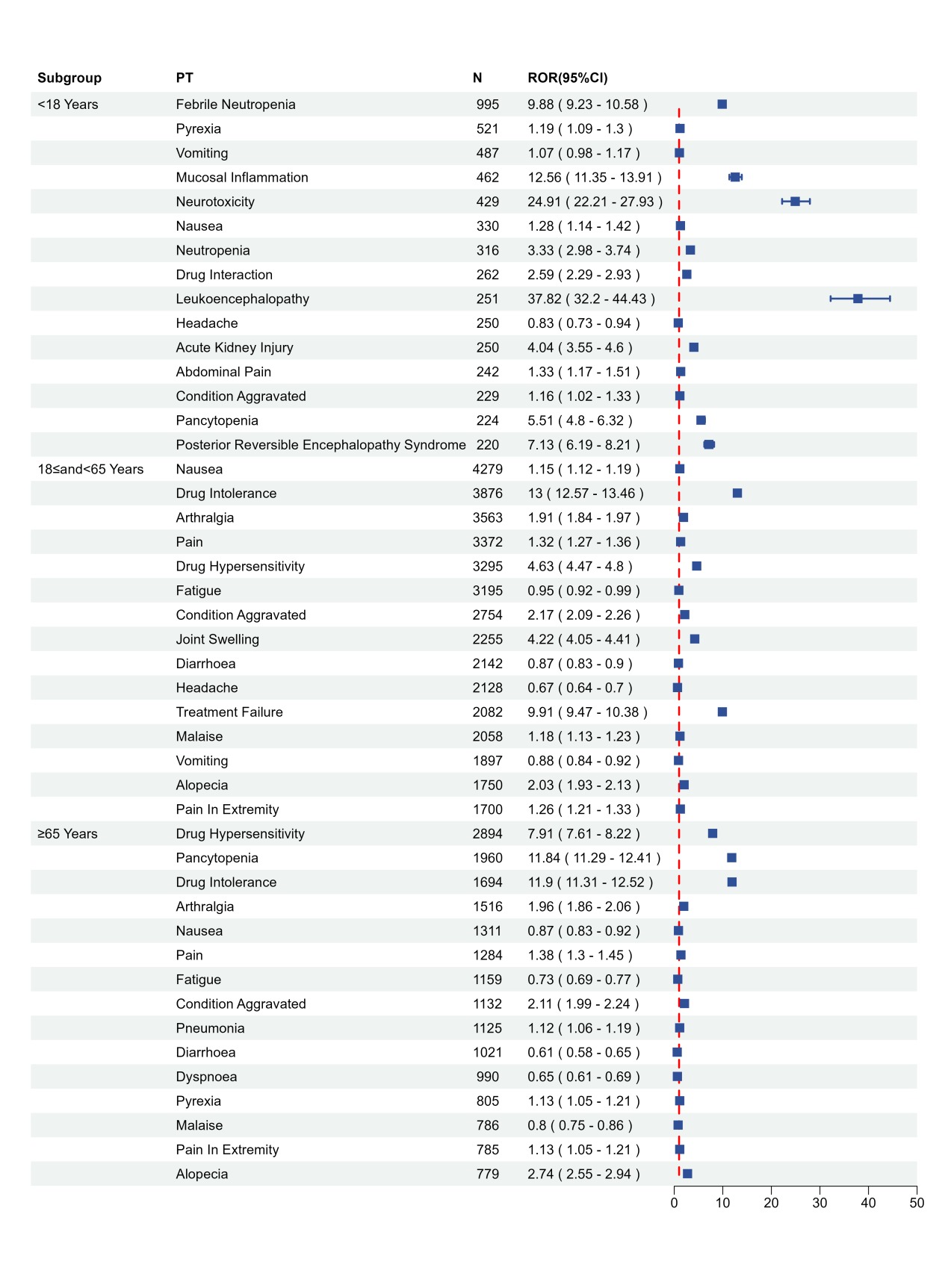


Supplementary Figure 4.The staff PT is ranked by ROR


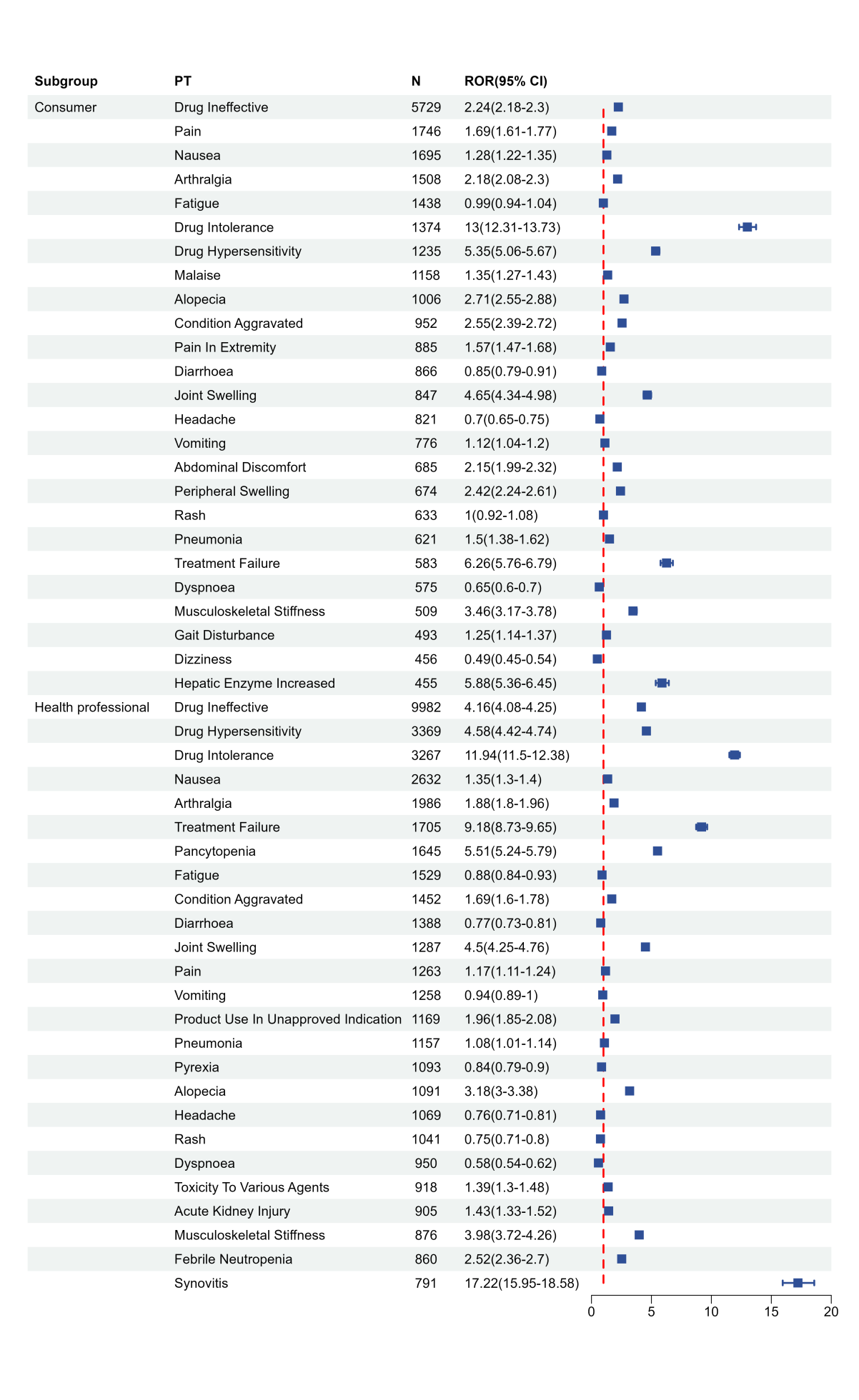


Supplementary Figure 5. Sex-induced time survival curve

Supplementary Figure 6.Age-induced time survival curve
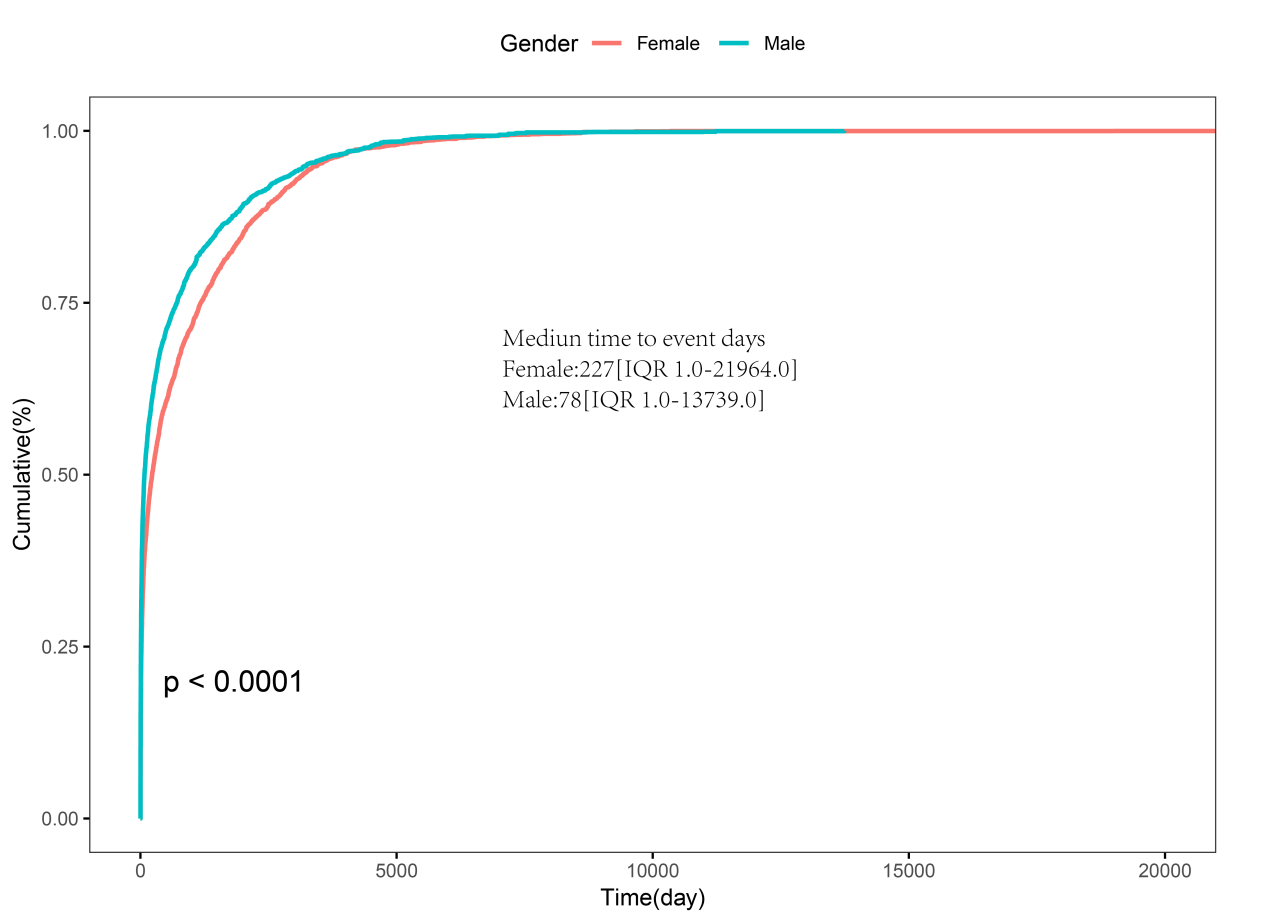


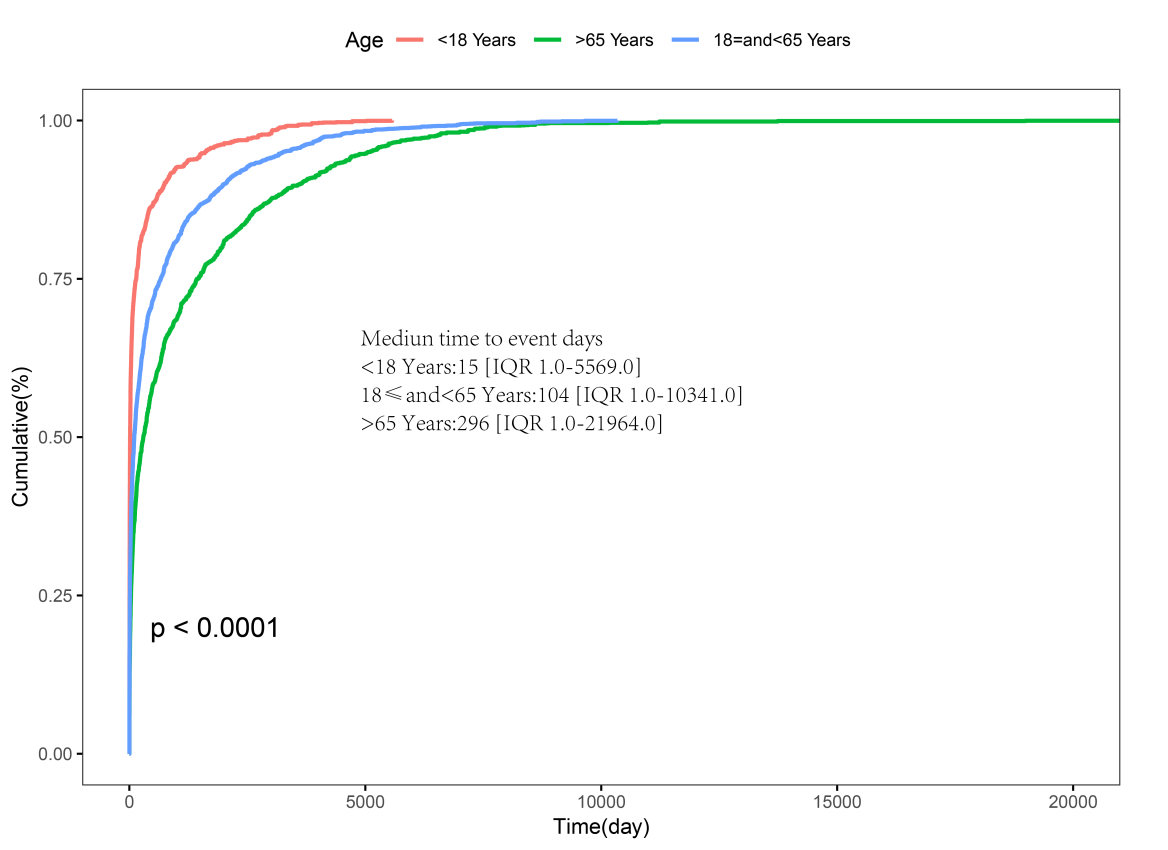


Supplementary Figure 7.Evoked time survival curve of reported population


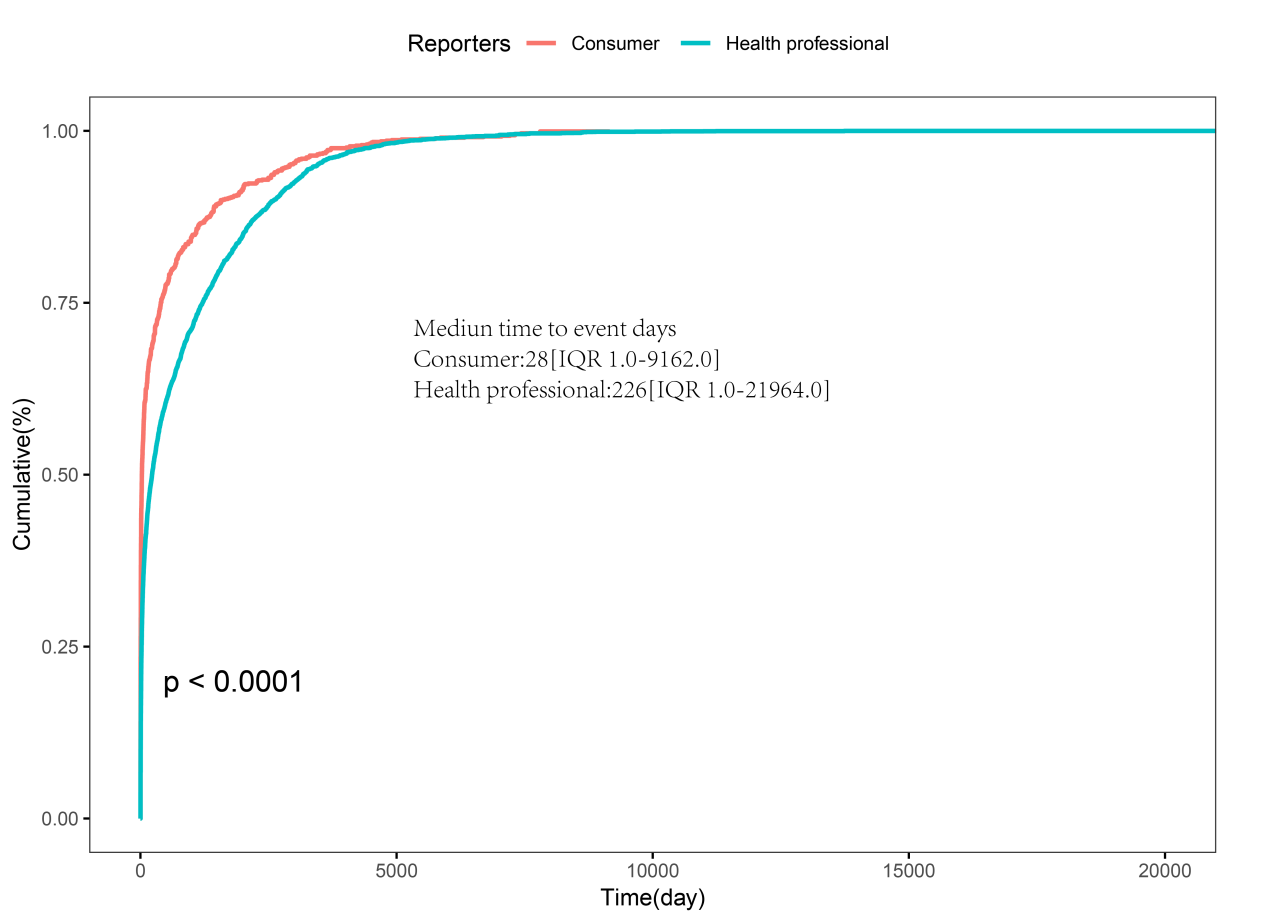


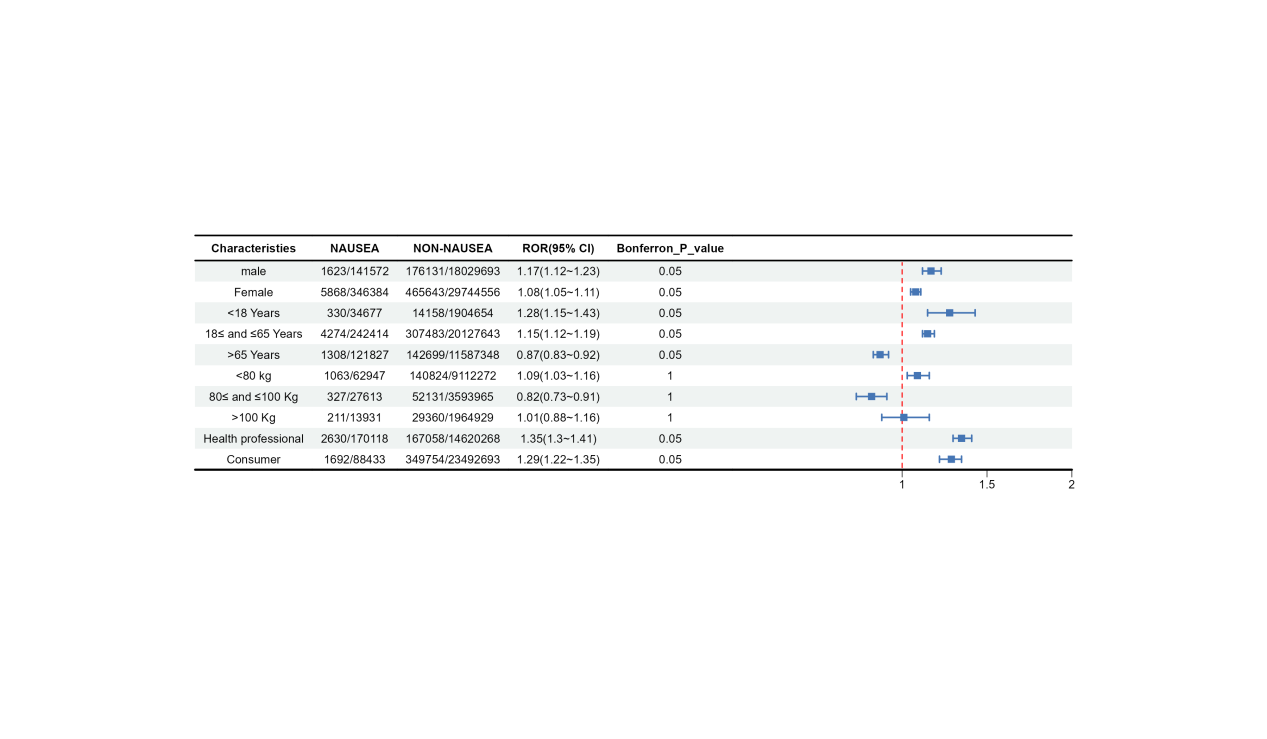
Supplementary Figure 8.Analysis of confounding factors such as gender, age and weight on nausea (AE)
