# Supplementary material for: Adverse drug reactions related to methotrexate: a real-world pharmacovigilance study using the FAERS database from 2004 to 2024
Source: Front Immunol. 2025 Jun 4;16:1586361. doi: 10.3389/fimmu.2025.1586361 (PMC12174053; doi:10.3389/fimmu.2025.1586361)
Supplement: Supplementary file 2 [file Table1.docx]

**Supplementary Table 1** Two-by-two contingency table for disproportionality analyses.

|  | Target AEs | Other AEs | Total |
| --- | --- | --- | --- |
| Target drugs | a | b | a+b |
| Other drugs | c | d | c+d |
| Total | a+c | b+d | a+b+c+d |

## Abbreviation: AEs, adverse events; a, number of reports containing both the target drug and target adverse drug reaction; b, number of reports containing other adverse drug reaction of the target drug; c, number of reports containing the target adverse drug reaction of other drugs; d, number of reports containing other drugs and other adverse drug reactions.

**Supplementary Table 2** Four major algorithms used for signal detection.

| Algorithms | Calculation Formula | Threshold |
| --- | --- | --- |
| ROR | $\begin{matrix} ROR=\frac{(a/c)}{(b/d)}=\frac{ad}{bc} \\ SE(InROR)=\sqrt{\left(\frac{1}{a}+\frac{1}{b}+\frac{1}{c}+\frac{1}{d}\right)} \\ 95\%CI=eIn(ROR)\pm1.96\sqrt{\left(\frac{1}{a}+\frac{1}{b}+\frac{1}{c}+\frac{1}{d}\right)} \end{matrix}$ | a≥3 with a lower 95% CI > 1 |
| PRR | $\begin{matrix} PRR=\frac{\text{a}/\left(a+b\right)}{c/(c+d)} \\ SE(InPRR)=\sqrt{\frac{1}{a}-\frac{1}{a+b}+\frac{1}{c}-\frac{1}{c+d}} \\ 95\%CI=eIn(PRR)\pm1.96\sqrt{(\frac{1}{a}-\frac{1}{a+b}+\frac{1}{c}-\frac{1}{c+d})} \end{matrix}$ | a≥3 with a lower 95% CI > 1 |
| BCPNN | α1=β1=1;α=β=2;γ11=1 | (-): E(IC)≤0  (+):0< E(IC) ≤1.5:  (++):1.5< E(IC) ≤3:  (+++):E(IC)>3 |
| MGPS |  | EBGM05>2,a>0 |

Abbreviation: a, number of reports containing both the target drug and target adverse drug reaction; b, number of reports containing other adverse drug reaction of the target drug; c, number of reports containing the target adverse drug reaction of other drugs; d, number of reports containing other drugs and other adverse drug reactions. 95%CI, 95% confidence interval; N, the number of reports; χ2, chi-squared; IC, information component; IC025, the lower limit of 95% CI of the IC; E(IC), the IC expectations; V(IC), the variance of IC; EBGM, empirical Bayesian geometric mean; EBGM05, the lower limit of 95% CI of EBGM.

**Supplementary Table 3** All adverse events meeting the positive signal threshold at the PT level from FAERS data**.**

| SOC | PT(Preferred Term) | a | ROR(95%Cl) | IC(IC025) | PRR(χ^2^) | EBGM(EBGM05) |
| --- | --- | --- | --- | --- | --- | --- |
| Blood And Lymphatic System Disorders | Pancytopenia | 3655 | 8.29 ( 8.02 - 8.58 ) | 2.95 ( 2.9 ) | 8.24 ( 21568.37 ) | 7.71 ( 7.49 ) |
|  | Febrile Neutropenia | 1940 | 3.54 ( 3.38 - 3.7 ) | 1.78 ( 1.72 ) | 3.53 ( 3402.17 ) | 3.44 ( 3.32 ) |
|  | Thrombocytopenia | 1588 | 1.67 ( 1.59 - 1.76 ) | 0.73 ( 0.66 ) | 1.67 ( 421.52 ) | 1.66 ( 1.59 ) |
|  | Neutropenia | 1556 | 1.34 ( 1.27 - 1.41 ) | 0.41 ( 0.34 ) | 1.34 ( 130.04 ) | 1.33 ( 1.28 ) |
|  | Leukopenia | 1314 | 3.12 ( 2.95 - 3.29 ) | 1.61 ( 1.53 ) | 3.11 ( 1830.39 ) | 3.05 ( 2.91 ) |
| Cardiac Disorders | Pericarditis | 313 | 2.42 ( 2.17 - 2.71 ) | 1.26 ( 1.09 ) | 2.42 ( 255.95 ) | 2.39 ( 2.18 ) |
|  | Cardiotoxicity | 92 | 1.28 ( 1.04 - 1.57 ) | 0.35 ( 0.05 ) | 1.28 ( 5.56 ) | 1.28 ( 1.07 ) |
|  | Cardiopulmonary Failure | 55 | 1.58 ( 1.21 - 2.07 ) | 0.65 ( 0.27 ) | 1.58 ( 11.62 ) | 1.57 ( 1.26 ) |
|  | Aortic Valve Stenosis | 48 | 3.12 ( 2.34 - 4.15 ) | 1.61 ( 1.19 ) | 3.12 ( 66.99 ) | 3.05 ( 2.4 ) |
|  | Sinus Node Dysfunction | 31 | 2.04 ( 1.43 - 2.91 ) | 1.02 ( 0.5 ) | 2.04 ( 16.17 ) | 2.02 ( 1.5 ) |
| Congenital, Familial And Genetic Disorders | Craniosynostosis | 67 | 9.2 ( 7.16 - 11.81 ) | 3.09 ( 2.73 ) | 9.2 ( 449.54 ) | 8.53 ( 6.92 ) |
|  | Foetal Methotrexate Syndrome | 56 | 484.25 ( 259.59 - 903.33 ) | 6.43 ( 5.91 ) | 484.2 ( 4765.27 ) | 86.27 ( 51.2 ) |
|  | Clinodactyly | 49 | 23.98 ( 17.58 - 32.72 ) | 4.3 ( 3.85 ) | 23.98 ( 876.54 ) | 19.67 ( 15.16 ) |
|  | Talipes | 46 | 1.64 ( 1.23 - 2.2 ) | 0.71 ( 0.28 ) | 1.64 ( 11.33 ) | 1.63 ( 1.28 ) |
|  | Cytogenetic Abnormality | 46 | 4.68 ( 3.48 - 6.29 ) | 2.18 ( 1.75 ) | 4.68 ( 127.33 ) | 4.52 ( 3.53 ) |
| Ear And Labyrinth Disorders | Ear Pain | 230 | 1.38 ( 1.21 - 1.57 ) | 0.45 ( 0.26 ) | 1.38 ( 23.27 ) | 1.37 ( 1.23 ) |
|  | Deafness Neurosensory | 62 | 2.3 ( 1.79 - 2.95 ) | 1.18 ( 0.81 ) | 2.3 ( 44.41 ) | 2.27 ( 1.84 ) |
|  | Sudden Hearing Loss | 33 | 2 ( 1.42 - 2.82 ) | 0.98 ( 0.48 ) | 2 ( 16.13 ) | 1.98 ( 1.48 ) |
|  | Deafness Transitory | 28 | 3.72 ( 2.55 - 5.43 ) | 1.86 ( 1.31 ) | 3.72 ( 53.87 ) | 3.63 ( 2.65 ) |
|  | Tympanic Membrane Perforation | 28 | 1.93 ( 1.33 - 2.81 ) | 0.94 ( 0.4 ) | 1.93 ( 12.4 ) | 1.92 ( 1.4 ) |
| Endocrine Disorders | Cushingoid | 94 | 3.58 ( 2.91 - 4.4 ) | 1.8 ( 1.5 ) | 3.58 ( 168.78 ) | 3.49 ( 2.94 ) |
|  | Cushing's Syndrome | 63 | 1.8 ( 1.41 - 2.31 ) | 0.84 ( 0.48 ) | 1.8 ( 22.16 ) | 1.79 ( 1.45 ) |
|  | Secondary Adrenocortical Insufficiency | 20 | 1.69 ( 1.08 - 2.62 ) | 0.74 ( 0.11 ) | 1.69 ( 5.49 ) | 1.67 ( 1.16 ) |
|  | Growth Hormone Deficiency | 10 | 2.91 ( 1.55 - 5.45 ) | 1.51 ( 0.63 ) | 2.91 ( 12.16 ) | 2.85 ( 1.69 ) |
|  | Delayed Puberty | 8 | 3.59 ( 1.78 - 7.27 ) | 1.81 ( 0.83 ) | 3.59 ( 14.47 ) | 3.51 ( 1.94 ) |
| Eye Disorders | Cataract | 739 | 1.49 ( 1.38 - 1.6 ) | 0.57 ( 0.46 ) | 1.49 ( 116.88 ) | 1.48 ( 1.39 ) |
|  | Uveitis | 467 | 4.3 ( 3.92 - 4.71 ) | 2.06 ( 1.92 ) | 4.29 ( 1133.59 ) | 4.16 ( 3.85 ) |
|  | Glaucoma | 251 | 1.52 ( 1.34 - 1.72 ) | 0.59 ( 0.41 ) | 1.52 ( 43.52 ) | 1.51 ( 1.36 ) |
|  | Iridocyclitis | 97 | 3.93 ( 3.21 - 4.82 ) | 1.94 ( 1.64 ) | 3.93 ( 204.29 ) | 3.82 ( 3.23 ) |
|  | Keratitis | 72 | 2.92 ( 2.31 - 3.69 ) | 1.52 ( 1.18 ) | 2.92 ( 88.16 ) | 2.86 ( 2.35 ) |
| Gastrointestinal Disorders | Nausea | 7762 | 1.15 ( 1.13 - 1.18 ) | 0.2 ( 0.17 ) | 1.15 ( 155.34 ) | 1.15 ( 1.13 ) |
|  | Abdominal Discomfort | 2665 | 1.89 ( 1.82 - 1.97 ) | 0.9 ( 0.85 ) | 1.89 ( 1096.12 ) | 1.87 ( 1.81 ) |
|  | Stomatitis | 1945 | 3.86 ( 3.69 - 4.04 ) | 1.91 ( 1.84 ) | 3.85 ( 3955.23 ) | 3.75 ( 3.61 ) |
|  | Gastrointestinal Disorder | 1917 | 2.76 ( 2.64 - 2.89 ) | 1.44 ( 1.37 ) | 2.75 ( 2087.19 ) | 2.71 ( 2.61 ) |
|  | Mouth Ulceration | 1506 | 9.17 ( 8.69 - 9.66 ) | 3.08 ( 3.01 ) | 9.14 ( 10039.22 ) | 8.48 ( 8.12 ) |
| General Disorders And Administration Site Conditions | Drug Intolerance | 7737 | 10.5 ( 10.25 - 10.75 ) | 3.25 ( 3.21 ) | 10.35 ( 59543.06 ) | 9.51 ( 9.32 ) |
|  | Pain | 6391 | 1.19 ( 1.16 - 1.22 ) | 0.25 ( 0.21 ) | 1.19 ( 192.05 ) | 1.19 ( 1.16 ) |
|  | Condition Aggravated | 4829 | 1.96 ( 1.9 - 2.02 ) | 0.95 ( 0.91 ) | 1.95 ( 2205.21 ) | 1.93 ( 1.89 ) |
|  | Treatment Failure | 4278 | 6.68 ( 6.47 - 6.89 ) | 2.65 ( 2.61 ) | 6.63 ( 19254.78 ) | 6.29 ( 6.13 ) |
|  | Pyrexia | 3361 | 1.12 ( 1.08 - 1.16 ) | 0.16 ( 0.11 ) | 1.12 ( 40.56 ) | 1.12 ( 1.08 ) |
| Hepatobiliary Disorders | Liver Disorder | 1019 | 2.74 ( 2.57 - 2.91 ) | 1.43 ( 1.34 ) | 2.73 ( 1092.42 ) | 2.69 ( 2.55 ) |
|  | Liver Injury | 879 | 4.94 ( 4.62 - 5.29 ) | 2.25 ( 2.15 ) | 4.93 ( 2632.17 ) | 4.75 ( 4.49 ) |
|  | Hepatotoxicity | 797 | 4.42 ( 4.12 - 4.74 ) | 2.1 ( 1.99 ) | 4.41 ( 2018.29 ) | 4.27 ( 4.03 ) |
|  | Hepatic Steatosis | 642 | 4.14 ( 3.82 - 4.48 ) | 2 ( 1.89 ) | 4.13 ( 1466.19 ) | 4.01 ( 3.76 ) |
|  | Hepatitis | 622 | 2.89 ( 2.67 - 3.13 ) | 1.5 ( 1.39 ) | 2.89 ( 747.13 ) | 2.84 ( 2.65 ) |
| Immune System Disorders | Drug Hypersensitivity | 7662 | 4.69 ( 4.58 - 4.8 ) | 2.16 ( 2.13 ) | 4.63 ( 20964.36 ) | 4.48 ( 4.39 ) |
|  | Anaphylactic Reaction | 543 | 1.2 ( 1.1 - 1.3 ) | 0.26 ( 0.13 ) | 1.2 ( 17.62 ) | 1.2 ( 1.11 ) |
|  | Immunosuppression | 394 | 5.88 ( 5.31 - 6.51 ) | 2.49 ( 2.34 ) | 5.88 ( 1509.88 ) | 5.62 ( 5.16 ) |
|  | Immunodeficiency | 337 | 2.92 ( 2.62 - 3.26 ) | 1.52 ( 1.36 ) | 2.92 ( 414.64 ) | 2.87 ( 2.62 ) |
|  | Immune System Disorder | 238 | 2.13 ( 1.88 - 2.43 ) | 1.08 ( 0.89 ) | 2.13 ( 140.35 ) | 2.11 ( 1.9 ) |
| Infections And Infestations | Pneumonia | 3434 | 1.26 ( 1.21 - 1.3 ) | 0.32 ( 0.27 ) | 1.25 ( 176.11 ) | 1.25 ( 1.22 ) |
|  | Infection | 2270 | 1.89 ( 1.81 - 1.97 ) | 0.9 ( 0.84 ) | 1.89 ( 930.11 ) | 1.87 ( 1.81 ) |
|  | Nasopharyngitis | 2022 | 1.3 ( 1.24 - 1.36 ) | 0.37 ( 0.31 ) | 1.3 ( 135.79 ) | 1.29 ( 1.25 ) |
|  | Sepsis | 1588 | 1.66 ( 1.58 - 1.74 ) | 0.72 ( 0.64 ) | 1.65 ( 404.54 ) | 1.64 ( 1.58 ) |
|  | Lower Respiratory Tract Infection | 1221 | 3.38 ( 3.19 - 3.58 ) | 1.72 ( 1.64 ) | 3.37 ( 1974.05 ) | 3.3 ( 3.14 ) |
| Injury, Poisoning And Procedural Complications | Accidental Overdose | 559 | 1.88 ( 1.73 - 2.04 ) | 0.9 ( 0.77 ) | 1.88 ( 225.2 ) | 1.86 ( 1.74 ) |
|  | Wound | 374 | 1.63 ( 1.48 - 1.81 ) | 0.7 ( 0.55 ) | 1.63 ( 90.45 ) | 1.62 ( 1.49 ) |
|  | Joint Injury | 357 | 1.95 ( 1.76 - 2.17 ) | 0.95 ( 0.8 ) | 1.95 ( 162.01 ) | 1.93 ( 1.77 ) |
|  | Foot Fracture | 244 | 1.47 ( 1.3 - 1.67 ) | 0.55 ( 0.36 ) | 1.47 ( 36.15 ) | 1.46 ( 1.32 ) |
|  | Joint Dislocation | 173 | 1.95 ( 1.68 - 2.26 ) | 0.95 ( 0.73 ) | 1.95 ( 78.32 ) | 1.93 ( 1.7 ) |
| Investigations | Hepatic Enzyme Increased | 2152 | 3.88 ( 3.72 - 4.05 ) | 1.91 ( 1.85 ) | 3.87 ( 4417.59 ) | 3.77 ( 3.63 ) |
|  | C-Reactive Protein Increased | 1792 | 6.03 ( 5.75 - 6.32 ) | 2.52 ( 2.45 ) | 6.01 ( 7080.76 ) | 5.74 ( 5.51 ) |
|  | Liver Function Test Increased | 1668 | 10.6 ( 10.08 - 11.15 ) | 3.28 ( 3.2 ) | 10.57 ( 13116.54 ) | 9.68 ( 9.28 ) |
|  | Alanine Aminotransferase Increased | 951 | 1.78 ( 1.67 - 1.9 ) | 0.82 ( 0.72 ) | 1.78 ( 317.37 ) | 1.76 ( 1.67 ) |
|  | Red Blood Cell Sedimentation Rate Increased | 748 | 8.3 ( 7.7 - 8.94 ) | 2.95 ( 2.84 ) | 8.29 ( 4438.85 ) | 7.75 ( 7.28 ) |
| Metabolism And Nutrition Disorders | Hypercalcaemia | 209 | 1.98 ( 1.73 - 2.28 ) | 0.97 ( 0.77 ) | 1.98 ( 100.11 ) | 1.97 ( 1.75 ) |
|  | Hypoalbuminaemia | 167 | 2.61 ( 2.24 - 3.04 ) | 1.36 ( 1.14 ) | 2.61 ( 161.47 ) | 2.57 ( 2.26 ) |
|  | Obesity | 166 | 1.23 ( 1.05 - 1.43 ) | 0.29 ( 0.07 ) | 1.23 ( 6.83 ) | 1.22 ( 1.08 ) |
|  | Hypercholesterolaemia | 148 | 1.93 ( 1.64 - 2.27 ) | 0.94 ( 0.7 ) | 1.93 ( 65.42 ) | 1.92 ( 1.67 ) |
|  | Folate Deficiency | 122 | 14.18 ( 11.73 - 17.13 ) | 3.65 ( 3.38 ) | 14.18 ( 1314.42 ) | 12.59 ( 10.75 ) |
| Musculoskeletal And Connective Tissue Disorders | Arthralgia | 6880 | 1.98 ( 1.93 - 2.03 ) | 0.96 ( 0.93 ) | 1.97 ( 3224.78 ) | 1.95 ( 1.91 ) |
|  | Joint Swelling | 4173 | 4.21 ( 4.08 - 4.35 ) | 2.02 ( 1.98 ) | 4.19 ( 9748.44 ) | 4.06 ( 3.96 ) |
|  | Pain In Extremity | 3218 | 1.24 ( 1.2 - 1.29 ) | 0.31 ( 0.26 ) | 1.24 ( 149.05 ) | 1.24 ( 1.2 ) |
|  | Musculoskeletal Stiffness | 2737 | 3.69 ( 3.56 - 3.84 ) | 1.84 ( 1.79 ) | 3.68 ( 5167.23 ) | 3.59 ( 3.48 ) |
|  | Synovitis | 2112 | 16.01 ( 15.29 - 16.76 ) | 3.8 ( 3.74 ) | 15.95 ( 25654.93 ) | 13.96 ( 13.43 ) |
| Neoplasms Benign, Malignant And Unspecified (Incl Cysts And Polyps) | Lymphoproliferative Disorder | 955 | 59.69 ( 55.12 - 64.64 ) | 5.26 ( 5.15 ) | 59.58 ( 34943.99 ) | 38.21 ( 35.75 ) |
|  | Diffuse Large B-Cell Lymphoma | 648 | 11.66 ( 10.75 - 12.65 ) | 3.4 ( 3.28 ) | 11.65 ( 5672.83 ) | 10.58 ( 9.88 ) |
|  | Basal Cell Carcinoma | 461 | 3.5 ( 3.19 - 3.84 ) | 1.77 ( 1.63 ) | 3.49 ( 794.42 ) | 3.41 ( 3.16 ) |
|  | Lymphoma | 392 | 2.95 ( 2.67 - 3.26 ) | 1.53 ( 1.39 ) | 2.95 ( 491.89 ) | 2.9 ( 2.66 ) |
|  | Acute Myeloid Leukaemia | 346 | 2.65 ( 2.38 - 2.95 ) | 1.38 ( 1.23 ) | 2.65 ( 347.32 ) | 2.61 ( 2.39 ) |
| Nervous System Disorders | Neurotoxicity | 846 | 6.26 ( 5.84 - 6.71 ) | 2.57 ( 2.47 ) | 6.25 ( 3518.36 ) | 5.95 ( 5.61 ) |
|  | Leukoencephalopathy | 560 | 20.1 ( 18.36 - 22 ) | 4.09 ( 3.95 ) | 20.08 ( 8505.9 ) | 16.98 ( 15.75 ) |
|  | Encephalopathy | 459 | 2.22 ( 2.02 - 2.44 ) | 1.13 ( 1 ) | 2.22 ( 301.32 ) | 2.19 ( 2.03 ) |
|  | Posterior Reversible Encephalopathy Syndrome | 380 | 5.02 ( 4.53 - 5.56 ) | 2.27 ( 2.12 ) | 5.01 ( 1164.88 ) | 4.83 ( 4.43 ) |
|  | Dysstasia | 361 | 1.41 ( 1.27 - 1.57 ) | 0.49 ( 0.34 ) | 1.41 ( 42.68 ) | 1.41 ( 1.29 ) |
| Pregnancy, Puerperium And Perinatal Conditions | Ruptured Ectopic Pregnancy | 41 | 6.26 ( 4.56 - 8.57 ) | 2.57 ( 2.12 ) | 6.26 ( 170.75 ) | 5.96 ( 4.58 ) |
|  | Tubal Rupture | 9 | 27.47 ( 13.17 - 57.26 ) | 4.45 ( 3.44 ) | 27.46 ( 181.48 ) | 21.93 ( 11.86 ) |
|  | Chronic Villitis Of Unknown Etiology | 4 | 24.41 ( 8.21 - 72.56 ) | 4.32 ( 2.89 ) | 24.41 ( 72.71 ) | 19.95 ( 8.02 ) |
| Product Issues | Prosthetic Cardiac Valve Malfunction | 4 | 59.29 ( 17.36 - 202.54 ) | 5.25 ( 3.72 ) | 59.29 ( 145.87 ) | 38.09 ( 13.63 ) |
| Psychiatric Disorders | Sleep Disorder Due To General Medical Condition, Insomnia Type | 126 | 2.58 ( 2.16 - 3.08 ) | 1.34 ( 1.08 ) | 2.58 ( 118.61 ) | 2.54 ( 2.19 ) |
|  | Drug Use Disorder | 71 | 1.31 ( 1.04 - 1.65 ) | 0.38 ( 0.04 ) | 1.31 ( 5.12 ) | 1.31 ( 1.07 ) |
|  | Adjustment Disorder With Depressed Mood | 58 | 4.33 ( 3.33 - 5.63 ) | 2.07 ( 1.69 ) | 4.33 ( 142.56 ) | 4.2 ( 3.37 ) |
|  | Somatic Symptom Disorder | 29 | 3.16 ( 2.19 - 4.58 ) | 1.63 ( 1.1 ) | 3.16 ( 41.65 ) | 3.1 ( 2.28 ) |
|  | Substance-Induced Psychotic Disorder | 27 | 1.54 ( 1.05 - 2.26 ) | 0.62 ( 0.07 ) | 1.54 ( 5.08 ) | 1.53 ( 1.12 ) |
| Renal And Urinary Disorders | Acute Kidney Injury | 1767 | 1.38 ( 1.32 - 1.45 ) | 0.46 ( 0.39 ) | 1.38 ( 184.56 ) | 1.38 ( 1.32 ) |
|  | Renal Impairment | 881 | 1.24 ( 1.16 - 1.33 ) | 0.31 ( 0.21 ) | 1.24 ( 41.15 ) | 1.24 ( 1.17 ) |
|  | Nephropathy Toxic | 306 | 3.42 ( 3.05 - 3.84 ) | 1.74 ( 1.57 ) | 3.42 ( 507.7 ) | 3.34 ( 3.04 ) |
|  | Cystitis Haemorrhagic | 101 | 3.01 ( 2.47 - 3.67 ) | 1.56 ( 1.27 ) | 3.01 ( 131.64 ) | 2.95 ( 2.5 ) |
|  | Lupus Nephritis | 61 | 4.54 ( 3.51 - 5.86 ) | 2.13 ( 1.76 ) | 4.54 ( 161.16 ) | 4.39 ( 3.54 ) |
| Reproductive System And Breast Disorders | Breast Mass | 79 | 1.36 ( 1.09 - 1.7 ) | 0.44 ( 0.12 ) | 1.36 ( 7.55 ) | 1.36 ( 1.13 ) |
|  | Cervical Dysplasia | 50 | 2.68 ( 2.02 - 3.55 ) | 1.4 ( 0.99 ) | 2.68 ( 51.38 ) | 2.64 ( 2.09 ) |
|  | Genital Ulceration | 30 | 5.62 ( 3.89 - 8.11 ) | 2.43 ( 1.9 ) | 5.62 ( 108.05 ) | 5.38 ( 3.96 ) |
|  | Genital Erosion | 30 | 12.92 ( 8.84 - 18.88 ) | 3.54 ( 2.99 ) | 12.92 ( 293.29 ) | 11.6 ( 8.44 ) |
|  | Rectocele | 21 | 3.93 ( 2.54 - 6.08 ) | 1.94 ( 1.31 ) | 3.93 ( 44.26 ) | 3.83 ( 2.66 ) |
| Respiratory, Thoracic And Mediastinal Disorders | Interstitial Lung Disease | 1337 | 3.35 ( 3.18 - 3.54 ) | 1.71 ( 1.63 ) | 3.35 ( 2135.27 ) | 3.28 ( 3.13 ) |
|  | Lung Disorder | 1145 | 2.8 ( 2.64 - 2.97 ) | 1.46 ( 1.37 ) | 2.8 ( 1286.66 ) | 2.75 ( 2.62 ) |
|  | Oropharyngeal Pain | 985 | 1.24 ( 1.17 - 1.32 ) | 0.31 ( 0.22 ) | 1.24 ( 45.94 ) | 1.24 ( 1.18 ) |
|  | Pulmonary Fibrosis | 935 | 6.13 ( 5.74 - 6.55 ) | 2.55 ( 2.45 ) | 6.12 ( 3786.17 ) | 5.84 ( 5.52 ) |
|  | Respiratory Failure | 883 | 1.4 ( 1.31 - 1.49 ) | 0.48 ( 0.38 ) | 1.4 ( 98 ) | 1.39 ( 1.32 ) |
| Skin And Subcutaneous Tissue Disorders | Alopecia | 3658 | 2.16 ( 2.09 - 2.24 ) | 1.09 ( 1.04 ) | 2.16 ( 2229.93 ) | 2.13 ( 2.08 ) |
|  | Psoriasis | 2279 | 2.03 ( 1.94 - 2.11 ) | 1 ( 0.94 ) | 2.02 ( 1158.48 ) | 2 ( 1.93 ) |
|  | Skin Ulcer | 714 | 3.27 ( 3.03 - 3.52 ) | 1.68 ( 1.57 ) | 3.26 ( 1087.32 ) | 3.19 ( 3 ) |
|  | Skin Lesion | 529 | 2.31 ( 2.12 - 2.52 ) | 1.19 ( 1.06 ) | 2.31 ( 383.71 ) | 2.28 ( 2.12 ) |
|  | Blister | 520 | 1.1 ( 1.01 - 1.2 ) | 0.14 ( 0.01 ) | 1.1 ( 4.61 ) | 1.1 ( 1.02 ) |
| Social Circumstances | Loss Of Personal Independence In Daily Activities | 1259 | 3.35 ( 3.17 - 3.54 ) | 1.71 ( 1.63 ) | 3.34 ( 2005.86 ) | 3.27 ( 3.12 ) |
|  | Impaired Work Ability | 257 | 1.37 ( 1.21 - 1.55 ) | 0.45 ( 0.27 ) | 1.37 ( 25.03 ) | 1.36 ( 1.23 ) |
|  | Immobile | 109 | 3.01 ( 2.49 - 3.64 ) | 1.56 ( 1.28 ) | 3.01 ( 142.06 ) | 2.95 ( 2.52 ) |
|  | Walking Aid User | 95 | 1.71 ( 1.4 - 2.09 ) | 0.76 ( 0.47 ) | 1.71 ( 27.54 ) | 1.7 ( 1.43 ) |
|  | Wheelchair User | 58 | 1.51 ( 1.17 - 1.96 ) | 0.59 ( 0.21 ) | 1.51 ( 9.93 ) | 1.51 ( 1.21 ) |
| Surgical And Medical Procedures | Knee Arthroplasty | 411 | 2.43 ( 2.21 - 2.68 ) | 1.26 ( 1.12 ) | 2.43 ( 338.66 ) | 2.4 ( 2.21 ) |
|  | Hip Arthroplasty | 253 | 2.03 ( 1.79 - 2.3 ) | 1.01 ( 0.82 ) | 2.03 ( 129.43 ) | 2.01 ( 1.81 ) |
|  | Joint Arthroplasty | 66 | 4.37 ( 3.42 - 5.59 ) | 2.08 ( 1.72 ) | 4.37 ( 164.6 ) | 4.23 ( 3.45 ) |
|  | Arthrodesis | 49 | 5.82 ( 4.36 - 7.76 ) | 2.48 ( 2.06 ) | 5.82 ( 185.08 ) | 5.56 ( 4.37 ) |
|  | Intestinal Anastomosis | 24 | 5.39 ( 3.58 - 8.12 ) | 2.37 ( 1.78 ) | 5.39 ( 81.57 ) | 5.17 ( 3.67 ) |
| Vascular Disorders | Blood Pressure Fluctuation | 329 | 1.56 ( 1.4 - 1.74 ) | 0.63 ( 0.47 ) | 1.56 ( 65.14 ) | 1.55 ( 1.42 ) |
|  | Vasculitis | 201 | 2.02 ( 1.76 - 2.32 ) | 1 ( 0.79 ) | 2.02 ( 101.23 ) | 2 ( 1.78 ) |
|  | Hypertensive Crisis | 149 | 1.54 ( 1.31 - 1.81 ) | 0.61 ( 0.38 ) | 1.54 ( 27.67 ) | 1.53 ( 1.34 ) |
|  | Granulomatosis With Polyangiitis | 119 | 13.47 ( 11.13 - 16.3 ) | 3.59 ( 3.31 ) | 13.46 ( 1215.42 ) | 12.03 ( 10.26 ) |
|  | Raynaud's Phenomenon | 86 | 2.05 ( 1.65 - 2.54 ) | 1.02 ( 0.71 ) | 2.05 ( 45.26 ) | 2.03 ( 1.7 ) |

Abbreviation: ROR, reporting odds ratio; PRR, proportional reporting ratio; EBGM, empirical Bayesian geometric mean; EBGM05, the lower limit of the 95% CI of EBGM; IC, information component; IC025, the lower limit of the 95% CI of the IC; CI, confidence interval; PT,preferred term.

**Supplementary Table 4.**The top 30 concomitant drugs and their occurrence frequencies.

| Concomitant Drugs | n |
| --- | --- |
| Prednisone | 19763 |
| Enbrel | 17657 |
| Humira | 15194 |
| Sulfasalazine | 13727 |
| Folic Acid | 12926 |
| Leflunomide | 12887 |
| Actemra | 11878 |
| Methotrexate Sodium | 11392 |
| Hydroxychloroquine | 10633 |
| Rituximab | 10241 |
| Orencia | 9386 |
| Cosentyx | 8840 |
| Xeljanz | 8056 |
| Remicade | 7877 |
| Prednisolone | 6792 |
| Arava | 6628 |
| Hydroxychloroquine Sulfate | 5833 |
| Plaquenil | 5815 |
| Cytarabine | 5665 |
| Cyclophosphamide | 5635 |
| Vincristine | 5197 |
| Infliximab | 4699 |
| Simponi | 4692 |
| Cimzia | 4041 |
| Dexamethasone | 3943 |
| Adalimumab | 3599 |
| Etanercept | 3423 |
| Celebrex | 2947 |
| Rituxan | 2931 |
| Azathioprine | 2806 |

**Supplementary Table 5.**The frequency and statistical differences of the top 10PT between fatal and non-fatal occurred

| PTname | a | Fatal | NON__Fatal | chisq_stat | pvalue |
| --- | --- | --- | --- | --- | --- |
| Death | 1097 | 1097(90.66) | 113(9.34) | 12846.78 | ＜0.05 |
| Pancytopenia | 982 | 982(26.87) | 2673(73.13) | 2182.83 | ＜0.05 |
| Sepsis | 656 | 656(41.31) | 932(58.69) | 2826.54 | ＜0.05 |
| Toxicity To Various Agents | 614 | 614(21.04) | 2304(78.96) | 865.09 | ＜0.05 |
| Pneumonia | 538 | 538(15.74) | 2880(84.26) | 389.55 | ＜0.05 |
| Respiratory Failure | 465 | 465(52.66) | 418(47.34) | 2781.75 | ＜0.05 |
| Septic Shock | 461 | 461(56.22) | 359(43.78) | 3002.06 | ＜0.05 |
| Multiple Organ Dysfunction Syndrome | 427 | 427(78.78) | 115(21.22) | 4222.69 | ＜0.05 |
| Drug Ineffective | 413 | 413(1.45) | 28144(98.55) | 1460.67 | ＜0.05 |
| Acute Kidney Injury | 327 | 327(18.53) | 1438(81.47) | 349.96 | ＜0.05 |

1.Fatal: Death；2.NON_Fatal: Non-fatal situation；3.chisq_stat: Chi-Squared Test

**Supplementary Table 6.**The median time of adverse reactions in different SoCs

| Soc name | Median time |
| --- | --- |
| RENAL AND URINARY DISORDERS | 12 |
| SKIN AND SUBCUTANEOUS TISSUE DISORDERS | 27 |
| HEPATOBILIARY DISORDERS | 32 |
| BLOOD AND LYMPHATIC SYSTEM DISORDERS | 36 |
| INVESTIGATIONS | 52 |
| NERVOUS SYSTEM DISORDERS | 52 |
| GASTROINTESTINAL DISORDERS | 53 |
| PSYCHIATRIC DISORDERS | 54 |
| IMMUNE SYSTEM DISORDERS | 55.5 |
| METABOLISM AND NUTRITION DISORDERS | 64.5 |
| PRODUCT ISSUES | 70 |
| GENERAL DISORDERS AND ADMINISTRATION SITE CONDITIONS | 131 |
| EAR AND LABYRINTH DISORDERS | 223 |
| RESPIRATORY, THORACIC AND MEDIASTINAL DISORDERS | 224 |
| INJURY, POISONING AND PROCEDURAL COMPLICATIONS | 225 |
| VASCULAR DISORDERS | 225 |
| REPRODUCTIVE SYSTEM AND BREAST DISORDERS | 237.5 |
| CONGENITAL, FAMILIAL AND GENETIC DISORDERS | 280 |
| PREGNANCY, PUERPERIUM AND PERINATAL CONDITIONS | 280 |
| EYE DISORDERS | 304 |
| SOCIAL CIRCUMSTANCES | 353.5 |
| INFECTIONS AND INFESTATIONS | 424 |
| ENDOCRINE DISORDERS | 480 |
| CARDIAC DISORDERS | 560 |
| MUSCULOSKELETAL AND CONNECTIVE TISSUE DISORDERS | 591 |
| SURGICAL AND MEDICAL PROCEDURES | 795 |
| NEOPLASMS BENIGN, MALIGNANT AND UNSPECIFIED (INCL CYSTS AND POLYPS) | 1096 |

**Supplementary Table 7.**The frequencies of different SoCs are statistically analyzed on a quarterly basis

| Soc name | Q1 | Q2 | Q3 | Q4 |
| --- | --- | --- | --- | --- |
| Blood And Lymphatic System Disorders | 634 | 607 | 563 | 693 |
| Cardiac Disorders | 239 | 185 | 201 | 261 |
| Congenital, Familial And Genetic Disorders | 9 | 22 | 15 | 21 |
| Ear And Labyrinth Disorders | 28 | 31 | 26 | 34 |
| Endocrine Disorders | 24 | 13 | 20 | 25 |
| Eye Disorders | 133 | 79 | 82 | 125 |
| Gastrointestinal Disorders | 802 | 896 | 778 | 919 |
| General Disorders And Administration Site Conditions | 973 | 811 | 822 | 849 |
| Hepatobiliary Disorders | 386 | 325 | 332 | 371 |
| Immune System Disorders | 67 | 46 | 71 | 70 |
| Infections And Infestations | 1080 | 891 | 784 | 1047 |
| Injury, Poisoning And Procedural Complications | 426 | 452 | 433 | 496 |
| Investigations | 782 | 660 | 643 | 756 |
| Metabolism And Nutrition Disorders | 192 | 178 | 138 | 182 |
| Musculoskeletal And Connective Tissue Disorders | 637 | 466 | 398 | 519 |
| Neoplasms Benign, Malignant And Unspecified (Incl Cysts And Polyps) | 399 | 354 | 281 | 304 |
| Nervous System Disorders | 705 | 719 | 483 | 613 |
| Pregnancy, Puerperium And Perinatal Conditions | 16 | 14 | 17 | 30 |
| Product Issues | 17 | 37 | 35 | 25 |
| Psychiatric Disorders | 188 | 193 | 122 | 176 |
| Renal And Urinary Disorders | 217 | 227 | 196 | 264 |
| Reproductive System And Breast Disorders | 63 | 42 | 33 | 24 |
| Respiratory, Thoracic And Mediastinal Disorders | 541 | 568 | 537 | 584 |
| Skin And Subcutaneous Tissue Disorders | 337 | 380 | 293 | 360 |
| Social Circumstances | 11 | 11 | 7 | 5 |
| Surgical And Medical Procedures | 93 | 49 | 58 | 67 |
| Vascular Disorders | 180 | 159 | 135 | 171 |

Q1: The first quarter Q2: Second Quarter Q3: Third Quarter Q4: Fourth Quarter.

**Supplementary Table 7.**Based on the chi-square test analysis of the top 50 PT frequencies

| PT | a | ROR(95%Cl) | pvalue | chisq_stat | test_used | Bonferron_P_value |
| --- | --- | --- | --- | --- | --- | --- |
| Drug Ineffective | 28557 | 2.66 ( 2.63 - 2.69 ) | 0 | 27253.5016601967 | Chi-Square | 0 |
| Rheumatoid Arthritis | 7790 | 8.78 ( 8.58 - 8.99 ) | 0 | 48818.5715694525 | Chi-Square | 0 |
| Nausea | 7754 | 1.16 ( 1.13 - 1.18 ) | 1.95354199001795e-36 | 158.914496153574 | Chi-Square | 1.56205217521835E-32 |
| Drug Intolerance | 7719 | 10.5 ( 10.25 - 10.75 ) | 0 | 59426.8877707713 | Chi-Square | 0 |
| Drug Hypersensitivity | 7649 | 4.69 ( 4.59 - 4.8 ) | 0 | 20960.1408261263 | Chi-Square | 0 |
| Off Label Use | 7638 | 1.08 ( 1.06 - 1.11 ) | 2.24856039989172e-11 | 44.7413681432764 | Chi-Square | 1.79795E-07 |
| Arthralgia | 6793 | 1.96 ( 1.91 - 2.01 ) | 0 | 3085.23575062638 | Chi-Square | 0 |
| Pain | 6371 | 1.19 ( 1.16 - 1.22 ) | 2.53916595163983e-43 | 190.447321161654 | Chi-Square | 2.03032E-39 |
| Fatigue | 5630 | 0.85 ( 0.82 - 0.87 ) | 1.23705500677822e-35 | 155.246212747212 | Chi-Square | 9.89149E-32 |
| Condition Aggravated | 4810 | 1.96 ( 1.9 - 2.01 ) | 0 | 2187.46274184287 | Chi-Square | 0 |
| Treatment Failure | 4269 | 6.68 ( 6.48 - 6.89 ) | 0 | 19226.0558078408 | Chi-Square | 0 |
| Diarrhoea | 4246 | 0.78 ( 0.75 - 0.8 ) | 3.5145276446295e-61 | 272.335675314581 | Chi-Square | 2.81022E-57 |
| Joint Swelling | 4157 | 4.21 ( 4.08 - 4.34 ) | 0 | 9690.55965533884 | Chi-Square | 0 |
| Vomiting | 3846 | 0.97 ( 0.94 - 1 ) | 0.0363856098678378 | 4.37894040965186 | Chi-Square | 1 |
| Pancytopenia | 3655 | 8.32 ( 8.04 - 8.6 ) | 0 | 21638.7333346983 | Chi-Square | 0 |
| Alopecia | 3655 | 2.17 ( 2.1 - 2.24 ) | 0 | 2238.93798332949 | Chi-Square | 0 |
| Headache | 3649 | 0.67 ( 0.65 - 0.69 ) | 2.42900716152128e-127 | 576.270033692942 | Chi-Square | 1.9422E-123 |
| Malaise | 3595 | 0.94 ( 0.91 - 0.97 ) | 8.56152930122389e-05 | 15.4300649210814 | Chi-Square | 0.684579883 |
| Pneumonia | 3418 | 1.25 ( 1.21 - 1.3 ) | 2.62143739711154e-39 | 172.063287210704 | Chi-Square | 2.0961E-35 |
| Pyrexia | 3347 | 1.12 ( 1.08 - 1.15 ) | 3.7402906136432e-10 | 39.2439315370399 | Chi-Square | 2.99074E-06 |
| Rash | 3278 | 0.9 ( 0.87 - 0.93 ) | 2.60304662210485e-09 | 35.4602819896116 | Chi-Square | 2.0814E-05 |
| Product Use In Unapproved Indication | 3264 | 1.71 ( 1.65 - 1.77 ) | 1.62120956274928e-206 | 940.398705506429 | Chi-Square | 1.2963E-202 |
| Pain In Extremity | 3195 | 1.24 ( 1.19 - 1.28 ) | 1.14097611721589e-32 | 141.682630171504 | Chi-Square | 9.12325E-29 |
| Dyspnoea | 2951 | 0.6 ( 0.58 - 0.62 ) | 1.15548358621017e-168 | 766.283816492363 | Chi-Square | 9.2392E-165 |
| Toxicity To Various Agents | 2918 | 2.11 ( 2.03 - 2.19 ) | 0 | 1655.34498274982 | Chi-Square | 0 |
| Musculoskeletal Stiffness | 2727 | 3.69 ( 3.55 - 3.83 ) | 0 | 5137.50205843234 | Chi-Square | 0 |
| Abdominal Discomfort | 2662 | 1.9 ( 1.82 - 1.97 ) | 3.44089150788677e-241 | 1099.91816565986 | Chi-Square | 2.7513E-237 |
| Peripheral Swelling | 2462 | 1.92 ( 1.84 - 1.99 ) | 1.31027579872301e-230 | 1051.23746347292 | Chi-Square | 1.0477E-226 |
| Contraindicated Product Administered | 2349 | 10.15 ( 9.73 - 10.59 ) | 0 | 17575.9089638563 | Chi-Square | 0 |
| Psoriasis | 2274 | 2.03 ( 1.95 - 2.11 ) | 1.43004398660667e-253 | 1156.88585181978 | Chi-Square | 1.1435E-249 |
| Infection | 2268 | 1.89 ( 1.82 - 1.97 ) | 3.62144607115376e-205 | 934.192740631813 | Chi-Square | 2.8957E-201 |
| Cough | 2254 | 0.95 ( 0.91 - 0.99 ) | 0.0190535160105754 | 5.4965971502284 | Chi-Square | 1 |
| Hepatic Enzyme Increased | 2151 | 3.89 ( 3.73 - 4.06 ) | 0 | 4430.20018756553 | Chi-Square | 0 |
| Synovitis | 2111 | 16.04 ( 15.33 - 16.8 ) | 0 | 25697.1132472267 | Chi-Square | 0 |
| Asthenia | 2068 | 0.63 ( 0.61 - 0.66 ) | 1.2118518850177e-95 | 430.585521483248 | Chi-Square | 9.68997E-92 |
| Weight Decreased | 2023 | 0.84 ( 0.8 - 0.88 ) | 7.19110680049326e-15 | 60.5451220591839 | Chi-Square | 5.75001E-11 |
| Therapeutic Product Effect Incomplete | 2016 | 3.55 ( 3.4 - 3.71 ) | 0 | 3559.00190855986 | Chi-Square | 0 |
| Nasopharyngitis | 1998 | 1.29 ( 1.23 - 1.34 ) | 6.76211379965023e-29 | 124.43612735854 | Chi-Square | 5.40699E-25 |
| Abdominal Pain | 1985 | 1 ( 0.96 - 1.04 ) | 0.966176270583317 | 0.00179813848321187 | Chi-Square | 1 |
| Stomatitis | 1944 | 3.87 ( 3.69 - 4.05 ) | 0 | 3965.76176058583 | Chi-Square | 0 |
| Arthritis | 1939 | 2.92 ( 2.79 - 3.05 ) | 0 | 2365.78761057783 | Chi-Square | 0 |
| Product Use Issue | 1916 | 1.24 ( 1.18 - 1.29 ) | 1.93641813333115e-20 | 85.8548304292397 | Chi-Square | 1.54836E-16 |
| Febrile Neutropenia | 1914 | 3.5 ( 3.34 - 3.66 ) | 0 | 3289.05038296877 | Chi-Square | 0 |
| Gastrointestinal Disorder | 1913 | 2.76 ( 2.64 - 2.89 ) | 0 | 2084.14720232067 | Chi-Square | 0 |
| Psoriatic Arthropathy | 1845 | 6.06 ( 5.78 - 6.35 ) | 0 | 7341.24950127592 | Chi-Square | 0 |
| Mucosal Inflammation | 1840 | 8.97 ( 8.55 - 9.4 ) | 0 | 11941.7128474621 | Chi-Square | 0 |
| Drug Interaction | 1795 | 1.31 ( 1.25 - 1.37 ) | 3.55534910207522e-30 | 130.281848849688 | Chi-Square | 2.84286E-26 |
| C-Reactive Protein Increased | 1792 | 6.05 ( 5.76 - 6.34 ) | 0 | 7103.73677538676 | Chi-Square | 0 |
| Dizziness | 1786 | 0.41 ( 0.39 - 0.43 ) | 9.88131291682493e-324 | 1479.62771393707 | Chi-Square | 7.9011E-320 |
| Gait Disturbance | 1783 | 1.06 ( 1.02 - 1.12 ) | 0.00876862485689515 | 6.86935592610108 | Chi-Square | 1 |

PT：Preferred Term；Check if there is an expected frequency less than 5 and decide which Test to use. If the expected frequency is less than 5, use Fisher's Exact Test p_value; Otherwise, use chisq_stat p_value.
